# TIGIT Marks Exhausted T Cells, Correlates with Disease Progression, and Serves as a Target for Immune Restoration in HIV and SIV Infection

**DOI:** 10.1371/journal.ppat.1005349

**Published:** 2016-01-07

**Authors:** Glen M. Chew, Tsuyoshi Fujita, Gabriela M. Webb, Benjamin J. Burwitz, Helen L. Wu, Jason S. Reed, Katherine B. Hammond, Kiera L. Clayton, Naoto Ishii, Mohamed Abdel-Mohsen, Teri Liegler, Brooks I. Mitchell, Frederick M. Hecht, Mario Ostrowski, Cecilia M. Shikuma, Scott G. Hansen, Mark Maurer, Alan J. Korman, Steven G. Deeks, Jonah B. Sacha, Lishomwa C. Ndhlovu

**Affiliations:** 1 Hawaii Center for HIV/AIDS, Department of Tropical Medicine, John A. Burns School of Medicine, University of Hawaii, Honolulu, Hawaii, United States of America; 2 Department of Microbiology and Immunology, Tohoku University Graduate School of Medicine, Sendai, Japan; 3 Vaccine and Gene Therapy Institute, Oregon Health and Science University, Portland, Oregon, United States of America; 4 Oregon National Primate Research Center, Oregon Health and Science University, Portland, Oregon, United States of America; 5 Department of Immunology, University of Toronto, Toronto, Ontario, Canada; 6 Division of Experimental Medicine, Department of Medicine, San Francisco General Hospital, University of California, San Francisco, San Francisco, California, United States of America; 7 HIV/AIDS Division, Department of Medicine, San Francisco General Hospital, University of California, San Francisco, San Francisco, California, United States of America; 8 Biologics Discovery California, Bristol-Myers Squibb, Redwood City, California, United States of America; Emory University, UNITED STATES

## Abstract

HIV infection induces phenotypic and functional changes to CD8^+^ T cells defined by the coordinated upregulation of a series of negative checkpoint receptors that eventually result in T cell exhaustion and failure to control viral replication. We report that effector CD8^+^ T cells during HIV infection in blood and SIV infection in lymphoid tissue exhibit higher levels of the negative checkpoint receptor TIGIT. Increased frequencies of TIGIT^+^ and TIGIT^+^ PD-1^+^ CD8^+^ T cells correlated with parameters of HIV and SIV disease progression. TIGIT remained elevated despite viral suppression in those with either pharmacological antiretroviral control or immunologically in elite controllers. HIV and SIV-specific CD8^+^ T cells were dysfunctional and expressed high levels of TIGIT and PD-1. *Ex-vivo* single or combinational antibody blockade of TIGIT and/or PD-L1 restored viral-specific CD8^+^ T cell effector responses. The frequency of TIGIT^+^ CD4^+^ T cells correlated with the CD4^+^ T cell total HIV DNA. These findings identify TIGIT as a novel marker of dysfunctional HIV-specific T cells and suggest TIGIT along with other checkpoint receptors may be novel curative HIV targets to reverse T cell exhaustion.

## Author Summary

HIV-1 infection contributes substantially to global morbidity and mortality, with no immediate promise of an effective vaccine. One major obstacle to vaccine development and therapy is to understand why HIV-1 replication persists in a person despite the presence of viral specific immune responses. The emerging consensus has been that these immune cells are functionally ‘exhausted’ or anergic, and thus, although they can recognize HIV-1 specific target cells, they are unable to effectively keep up with rapid and dynamic viral replication in an individual. We have identified a novel combination pathway that can be targeted, TIGIT and PD-L1which may be responsible, at least in part, for making these immune cells dysfunctional and exhausted and thus unable to control the virus. We show that by blocking the TIGIT and PD-L1 pathway, we can reverse the defects of these viral specific immune cells. Our findings will give new directions to vaccines and therapies that will potentially reverse these dysfunctional cells and allow them to control HIV-1 replication, but also serve in “Shock and Kill” HIV curative strategies.

## Introduction

During chronic viral infections, high antigenic loads continually stimulate T cells leading to progressive loss of function termed “T cell exhaustion” [1]. Throughout this period, T cells increase expression of several inhibitory immune receptors that raise the threshold for activation, resulting in suppressed immune responses. While Programmed Death Receptor-1 (PD-1) was one of the earliest surface markers of immune exhaustion identified [2–7], we have shown that the surface glycoprotein, T cell immunoglobulin- and mucin domain-containing molecule (Tim)-3, defines a state of T cell exhaustion with diminished proliferative and cytokine capacities in chronic viral infection [8,9]. Thus, the upregulation of these and other negative checkpoints receptors may serve as potential targets for the reversal of T cell exhaustion.

Indeed, blocking the interaction of T cell negative checkpoint receptor pathways using targeted reagents against PD-1/Programmed Death-Ligand 1 (PD-L1), Tim-3, Lymphocyte-activation gene 3 (Lag-3) and CD160 has shown promise in reversing CD8^+^ T cell exhaustion [7,8,10–12]. Reagents targeting many of these receptors are rapidly advancing in the clinic and are showing efficacy in the control of viral infectious disease [13] as well as anti-tumor immunity [14–19]. A single dose of an antibody against PD-1 led to sustained clearance of hepatitis C virus infection in a small subset of individuals [13]. Blockade of the PD-1/PD-L1 axis *in vivo* demonstrated efficacy in restoring simian immunodeficiency virus (SIV)-specific T cell and humoral immunity, and led to a reduction of SIV viremia and in immune activation. However, this did not completely control virus, suggesting that additional therapies are needed. Importantly, not all features of the exhausted T-cells are restored by interfering with single pathways [2–4,8,20]. Synergistic simultaneous dual blockade has yielded more promising responses suggesting these co-inhibitory molecules are non-redundant [10,19,21,22].

T cell immunoreceptor with immunoglobulin and ITIM domains (TIGIT) is a recently described immune checkpoint receptor that belongs to the CD28 family and contains an extracellular IgV domain, a transmembrane domain, and a cytoplasmic tail containing two-immunoreceptor tyrosine-based inhibitory motif (ITIM) [23]. It has been reported to be expressed on natural killer (NK) cells, CD8^+^ T cells and CD4^+^ T cell subsets [23] and is induced upon activation [23–27]. TIGIT competes with DNAM-1, a co-stimulatory molecule, and TACTILE, a co-inhibitory molecule, for the poliovirus receptor (PVR) a member of the nectin family of adhesion molecules that is expressed on dendritic cells (DCs) [23,24,28]. Several murine and human studies strongly suggest that TIGIT is a negative modulator of T cell and NK cell function [25,29–31]. A number of plausible mechanisms exist by which TIGIT can mediate inhibition of T and NK cell activation. Signaling through the TIGIT/PVR pathway with the standard recruitment of phosphatases via the intracellular ITIM domain of TIGIT can curtail T cell and NK cell responses [26]. This interaction has been shown to induce tolerogenic DCs to release the immunosuppressive cytokine IL-10 [25,31]. Furthermore, disruption of DNAM-1 homodimerization by TIGIT can abrogate the positive co-stimulatory signals required for activation [18]. Recently, potent anti-viral and anti-tumor responses related to enhanced CD8^+^ T cell effector activity were generated following synergistic dual blockade of PD-L1 and TIGIT in the mouse model of chronic lymphocytic choriomeningitis virus (LCMV) infection [18] and *ex-vivo* in patients with advanced melanoma [19].

To date, these results have not been replicated in any human viral disease, but over-expression of both TIGIT and PD-1 on virally exhausted T cells suggests that this is a promising avenue of exploration as a viable strategy to increase control or eliminate viral infections through T cell modulation. Given the potential to restore anti-HIV-specific CD8^+^ T cell responses by synergistic modulation of negative checkpoint receptors, we investigated the expression and function of TIGIT in HIV disease pathogenesis, and in the SIV non-human primate model of HIV/AIDS.

## Results

### Expansion of TIGIT^+^ T cells during HIV infection and correlations with clinical parameters of HIV disease progression, T cell activation, and the CD4^+^ cell-associated HIV DNA

**Fig 1:**
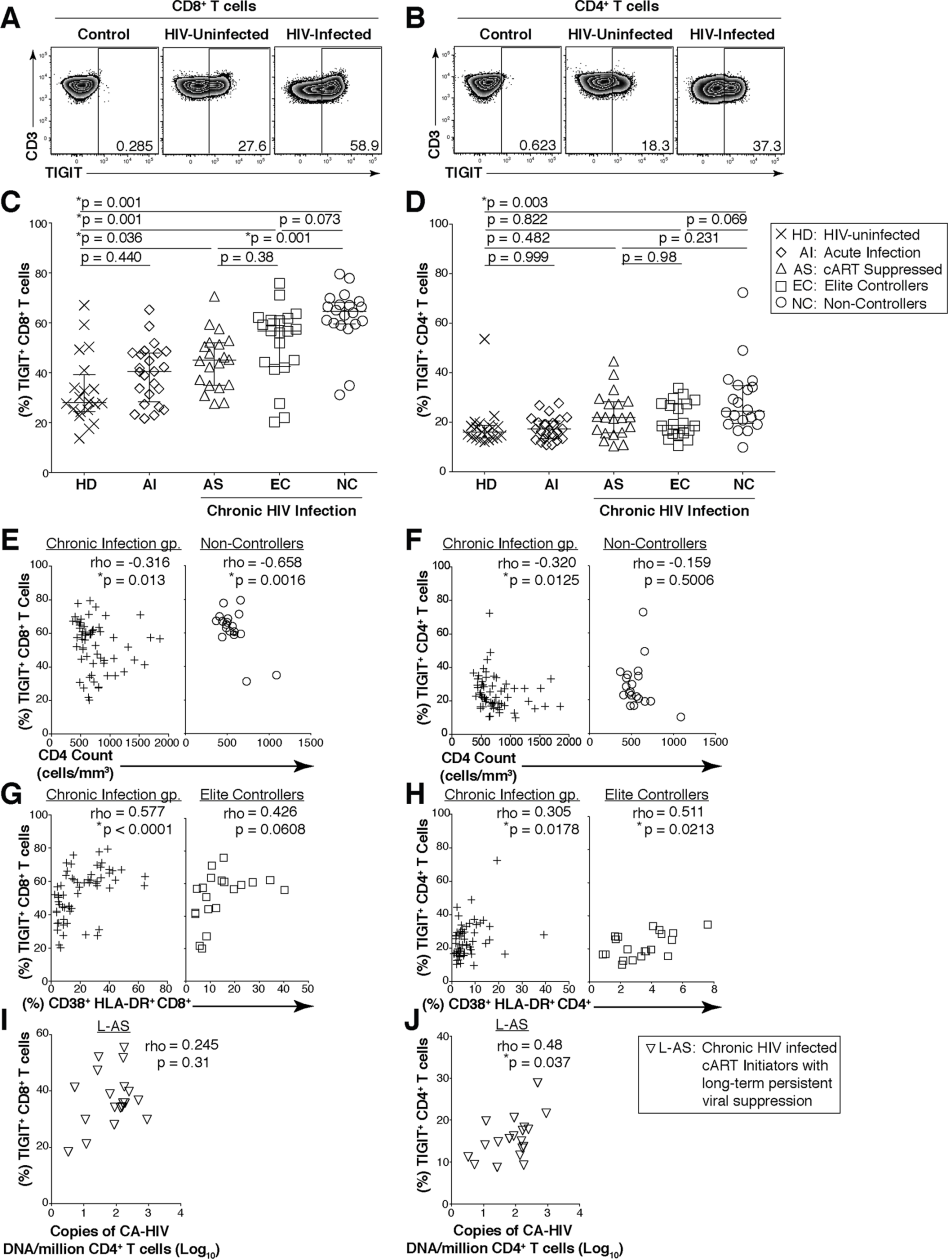
Expression of TIGIT on T cells during HIV infection. Cryopreserved PBMCs were thawed and surface phenotyped for TIGIT expression. Representative flow cytometry flow plots showing TIGIT expression on (A) CD8^+^ or (B) CD4^+^ T cells compared to fluorescence minus one (FMO) control. Graphs show compiled data of TIGIT expression on (C) CD8^+^ and (D) CD4^+^ T cells stratified by disease: HIV-uninfected healthy donors (HD, X; *n* = 20), acute HIV-infection (AI, open diamond; *n* = 24), aviremic cART suppressed (AS, open triangles; *n* = 20), aviremic elite controllers (EC, open squares; *n* = 20), and chronic HIV viremic non-controllers (NC, open circles; *n* = 20). P values were calculated using one-way ANOVA, followed by Tukey’s multiple comparisons test. Graphs show correlation of total chronic infected (+: AS, EC, and NC; left panel, *n* = 60) and non-controllers (right panel, *n* = 20) frequency (%) of (E) TIGIT^+^ CD8^+^ and (F) TIGIT^+^ CD4^+^ T cells against clinical CD4 Count (cells/mm^3^). Graphs show correlation of total chronic infected (+: AS, EC, and NC; left panel, *n* = 60) and elite controllers (right panel, *n* = 20) frequency (%) of (G) TIGIT^+^ CD8^+^ and (H) TIGIT^+^ CD4^+^ T cells against frequency (%) of T cell activation (CD38^+^HLA-DR^+^). Graphs show correlation of frequency (%) of (I) TIGIT^+^ CD8^+^ and (J) TIGIT^+^ CD4^+^ T cell among aviremic HIV infected “ART initiators” with known duration of long-term viral suppression from the SCOPE cohort (L-AS, *n* = 19, open inverted triangles) versus copies of CD4^+^ T cell associated HIV DNA per million CD4^+^ T cells (log_10_). Spearman’s rho tests were performed for correlations.

We assessed the surface expression of TIGIT on T cells from peripheral blood mononuclear cells (PBMCs) from HIV-infected individuals that were either acutely infected (AI), non-controllers (NC), cART suppressed (AS), or elite controllers (EC), and compared these results to age-matched HIV-uninfected healthy donors (HD) (Table 1; Figs 1A–1D, S1A and S1B). We observed a significant expansion in the frequency of TIGIT^+^ CD8^+^ T cells in HIV-infected participants (AS; 44.95%; EC 56.7%; NC, 64.5%), even among those with viral suppression, relative to HD (median: 28.05%; Fig 1C). We observed a non-significant trend in the expansion of TIGIT^+^ CD8^+^ T cells in AI (40.4%) relative to HD (Fig 1C). TIGIT^+^ CD4^+^ T cells were significantly elevated among NC (24.5%) compared to HD (16.05%) (Fig 1D).

Among the HIV-infected NC, TIGIT^+^ CD8^+^ T cells inversely correlated with CD4 cell counts, but not with CD8^+^ T cell activation or plasma viral load (Figs 1E and S1E). TIGIT^+^ CD4^+^ T cells did not correlate with CD4 cell counts in NC (Fig 1F). Among EC, TIGIT^+^ CD8^+^ T cells trended to correlate with CD8^+^ T cell activation, while frequencies of TIGIT^+^ CD4^+^ T cells correlated with CD4^+^ T cell activation (Fig 1G and 1H). We did not observe any other significant correlations with TIGIT^+^ T cells (S1C–S1F,S1G and S1I Fig). Given the high levels of TIGIT in the midst of viral suppression, we assessed the relationship between TIGIT and the cellular HIV content in highly purified CD4^+^ T cells among HIV-infected “cART initiators” who met strict selection criteria of well documented and long-term persistent viral suppression (L-AS; Table 1). We did not observe a correlation with the frequency of CD8^+^ T cell or CD4^+^ T cell TIGIT expression and HIV RNA from purified CD4^+^ T cells (S1H and S1J Fig). However, the frequency of TIGIT^+^ CD4^+^ T cells positively correlated with purified CD4^+^ T cell HIV DNA content, but not with frequency of TIGIT^+^ CD8^+^ T cells (Fig 1I and 1J). These data indicate that TIGIT expression on CD4^+^ T cells may be linked to chronic HIV disease pathogenesis, residual immune activation, and the cellular HIV DNA content among those with viral suppression.

**Table 1:** Participant characteristics.

	HIV+ Non-Controllers (NC; n = 20)	HIV+ Elite Controller (EC; n = 20)	HIV+ cART Suppressed (AS; n = 20)	HIV+ Known Duration of cART (L-AS; n = 19)	HIV+ Acute Infection (AI; n = 24)	HIV-Uninfected Donors (HD; n = 20)
Parameters	Median (IQR)	Median (IQR)	Median (IQR)	Median (IQR)	Median (IQR)	Median (IQR)
**Age** (Years)	44 (38, 55.5)	51 (44.5, 55.75)	50 (46, 57)	43 (37,51)	35 (32, 43)	50 (47, 55)
**HIV Viral Load** (copies/ml) Log10	4.51 (4.21, 5.05)	1.60 (1.60, 1.60)	1.60 (1.60, 1.87)	1.60 (1.60,1.60)	4.66 (2.28, 6.715)	N/A
**CD4+ T cell count** (cells/mm3)	518.5 (460, 626.5)	780.5 (659.5, 1248)	719.5 (542.8, 898.5)	519 (399,726)	640 (482. 3,790)	N/A
**CD4+ T cell nadir count** (cells/mm3)	371.5 (298.5, 439.3)	624.5 (376, 825.5)	115.5 (26.75, 260.8)	208 (92,275)	N/A	N/A
**CD4+ Activation** (% CD38+ HLA-DR+)	9.66 (8.22, 15.7)	3.46 (1.91, 4.60)	3.33 (2.82, 5.34)	7.26 (5.71,9.94)	3.58 (2.28, 6.71)	1.9 (1.65,2.68)
**CD8+ Activation** (% CD38+ HLA-DR+)	33.65 (28.53, 41.80)	10.45 (6.77, 18.63)	8.71 (5.12, 17.10)	20.7 (14.30,30.30)	20.1 (12.45, 34.83)	6.5 (4.27,11.15)
**CA-HIV DNA** (copies/million CD4 cells) Log10	N/A	N/A	N/A	2.13 (1.42,2.25)	N/A	N/A
**CA-HIV RNA** (copies/million CD4 cells) Log10	N/A	N/A	N/A	3.71 (2.93,4.28)	N/A	N/A
**Duration of Viral Suppression** (days)	N/A	N/A	N/A	784.5 (670.8,1034)	N/A	N/A
**Gender Distribution**						
**Male** % (no.)	80 (16)	80 (16)	75 (15)	70 (14)	91.6 (22)	90 (18)
**Female** % (no.)	5 (1)	15 (3)	15 (3)	25 (5)	8.3 (2)	10 (2)
**MTF** % (no.)	10 (2)	5 (1)	10 (2)	5 (1)	0 (0)	0 (0)
**Intersex** % (no.)	5 (1)	0 (0)	0 (0)	0 (0)	0 (0)	0 (0)

N/A = Not Available

CA: Cell Associated

### Phenotype and function of TIGIT on CD8^+^ T cells during HIV infection

**Fig 2:**
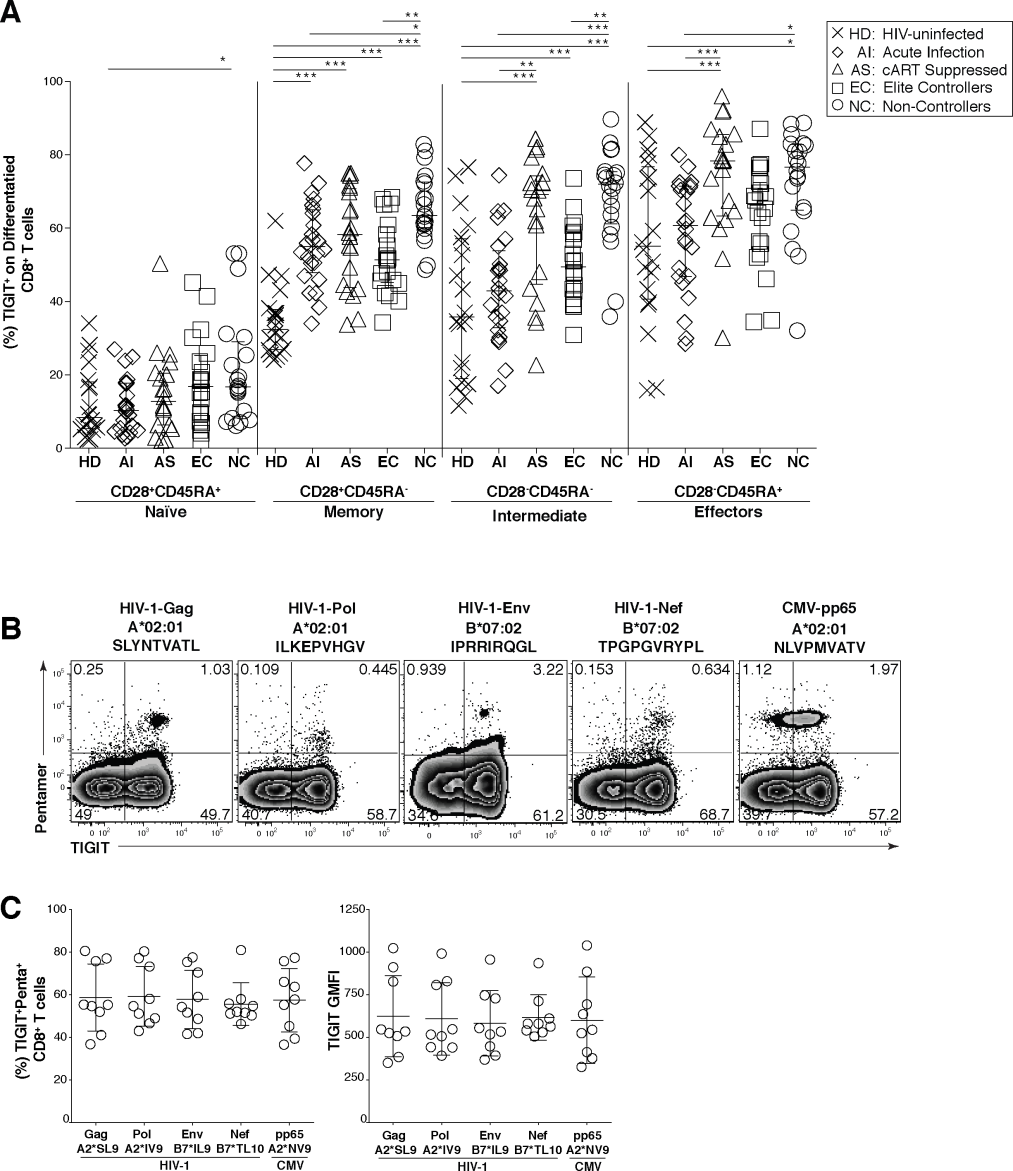
TIGIT expression on CD8^+^ terminal effector T cells and HIV-specific CD8^+^ T cells. Cryopreserved PBMCs were thawed and surface phenotyped for TIGIT expression on CD8^+^ T cell compartments. (A) Graph shows compiled frequency (%) of TIGIT^+^ CD8^+^ T cell expression in differentiated compartments stratified by disease status. HIV-uninfected healthy donors (HD, X; *n* = 20), acute infected (AI, open diamond; *n* = 24), cART suppressed (AS, open triangle; *n* = 20), elite controller (EC, open square; *n* = 20), non-controllers (NC, open circle; *n* = 20). P values were calculated using one-way ANOVA, followed by Tukey’s multiple comparisons test (*p < 0.05; **p < 0.01; ***p < 0.001). PBMCs from HLA-A*02:01 or HLA-B*07:02 HIV chronically infected individuals were stained with matched HLA pentamers presenting HIV-1 and CMV epitopes and anti-TIGIT. (B) Representative flow cytometry plots of pentamer-specific CD8^+^ T cells using HLA-A*02:01 HIV-1 Gag SLYNTVATL (A2*SL9), HLA-A*02:01 HIV-1 Pol ILKEPVHGV (A2*IV9), HLA-B*07:02 HIV-1 Env IPRRIRQGL (B7*IL9), HLA-B*07:02 HIV-1 Nef TPGPGVRYPL (B7*TL10), and HLA-A*02:01 CMV pp65 NLVPMVATV (A2*NV9) (C) Compiled data of TIGIT expression frequency (%) on pentamer specific CD8^+^ T cells which was recalculated to 100% (left panel, *n* = 9) compiled data of TIGIT geometric mean fluorescence intensity (GMFI) on pentamer specific CD8^+^ T cells (right panel, *n* = 9).

HIV infection leads to an expansion of intermediately differentiated memory CD8^+^ T cells that are not fully mature effectors [32–34]. We profiled the pattern of TIGIT expression in the heterogeneous CD8^+^ T cell subpopulations and found TIGIT was significantly expanded on the CD8^+^ T cell intermediate/transitional and effector subsets with the highest expression of TIGIT on the effector CD8^+^ T cell subset (Figs 2A and S2A–S2E) compared to AS. In the naïve population TIGIT expression was relatively stable with only a significant difference seen between HD and the non-controllers (Fig 2A). We did observe a statistically significant difference in TIGIT expression between the HD and AI group in the memory CD8^+^ T cell population (Fig 2A). Thus, TIGIT is expanded on the intermediate/transitional and effector CD8^+^ T cell subsets during chronic HIV infection, consistent with a role for TIGIT as potential regulator of intermediate/transitional and effector T cell responses.

We next profiled the expression of TIGIT on viral specific CD8^+^ T cells from chronically HIV-infected participants using matched HLA-I restricted pentamers for various HIV and CMV peptide epitopes. TIGIT was expressed on over half of all CD8^+^ T cells for specific for HIV-1 Gag (55.3%), Polymerase (54.7%), Envelope (54.3%), Nef (52%), and also for CMV pp65 (57.8%) (Fig 2B and 2C). Comparable levels of TIGIT on HIV and CMV specific CD8^+^ T cells were observed on a per cell basis as measured by Geometric Mean Fluorescence Intensity (GMFI) (Fig 2C).

**Fig 3:**
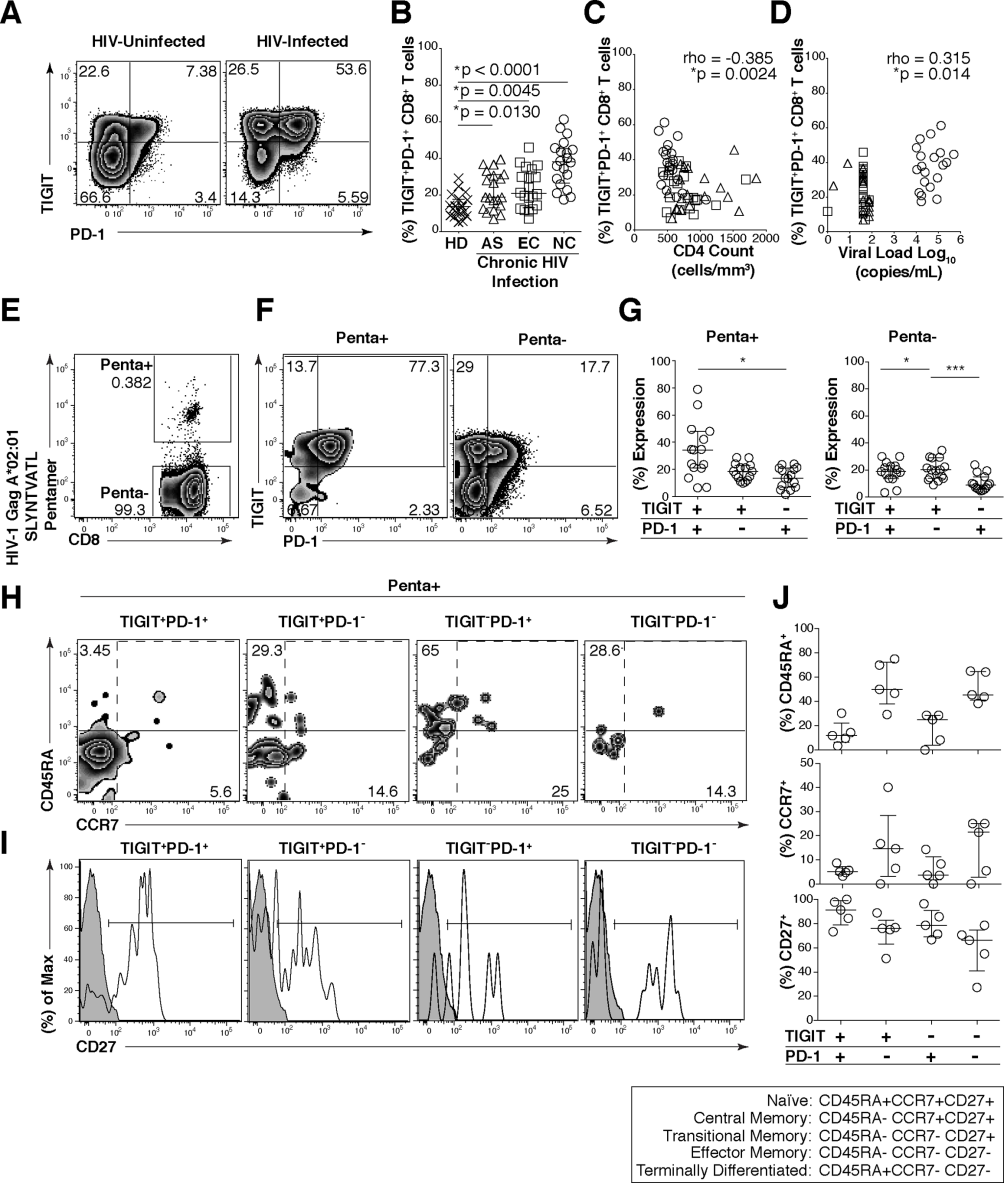
HIV-Gag specific CD8^+^ T cells co-express TIGIT and PD-1 and exhibit a transitional memory phenotype. Cryopreserved PBMCs were thawed and surface phenotyped for TIGIT and PD-1 expression on CD8^+^ T cells. (A) Representative flow cytometry plots showing TIGIT and PD-1 expression on CD8^+^ T cells from one HIV-uninfected individual (left panel) and one HIV-infected individual (right panel). (B) Graph shows compiled frequency (%) of co-expressing TIGIT^+^PD-1^+^ CD8^+^ T cells from HIV-uninfected (HD, *n* = 20), chronic HIV-infected (AS, *n* = 20; EC, *n* = 20; NC, *n* = 20). P values were calculated using one-way ANOVA, followed by Tukey’s multiple comparisons test. (C) Graph shows correlation of TIGIT^+^PD-1^+^ CD8^+^ T cells frequency (%) from chronic HIV-infected individuals against CD4 count (cells/mm^3^) or (D) viral load (copies/ml). Spearman’s rho tests were performed for correlations. TIGIT and PD-1 expression on HIV-1 Gag specific CD8^+^ T cells were evaluated. (E) Representative flow cytometry plot of HIV-specific CD8^+^ T cells using HLA-A*02:01 HIV-1 Gag SLYNTVATL. (F) Representative flow cytometry plots of TIGIT and PD-1 expression on HIV-1 Gag specific CD8^+^ T cells (Penta+, left panel; Penta-, right panel). (G) Graphs show compiled frequency (%) of TIGIT and PD-1 on Penta+ (left panel) and Penta- (right panel) (sample group contains; AS *n* = 11, EC *n* = 2, NC *n* = 2). P values were calculated using repeated-measures one-way ANOVA, followed by Tukey’s multiple comparisons test (*p < 0.05; **p < 0.01; ***p < 0.001). Representative flow cytometry of (H) CD45RA and CCR7 or (I) histogram of CD27 (shaded isotype control) on Penta+ CD8^+^ T cells expressing TIGIT^+^PD1^+^, TIGIT^+^PD-1^-^, TIGIT^-^PD-1^+^, or TIGIT^-^PD-1^-^. (J) Graphs show compiled frequency (%) of CD45RA (top panel), CCR7 (mid panel), and CD27 (bottom panel) (*n* = 5).

We next assessed the effector phenotype and functional properties of TIGIT expressing CD8^+^ T cells to determine whether they retain features of immune exhaustion. We found that most of the TIGIT expressing CD8^+^ T cells co-expressed PD-1 with the frequency of TIGIT^+^ PD-1^+^ CD8^+^ T cells significantly expanded in chronic HIV infection (AS, 18.65%; EC, 20.85%; NC, 38.15%) compared to HD (13.65%) (Fig 3A and 3B). The frequency of TIGIT^+^ PD-1^+^ CD8^+^ T cells inversely correlated with CD4 counts (Fig 3C) and positively correlated with plasma viral load levels (Fig 3D) among all chronically HIV-infected individuals. We observed significantly higher frequencies of TIGIT^+^PD-1^+^ co-expression on HIV-Gag-specific CD8^+^ T cells compared to non-HIV-Gag-specific CD8^+^ T cells derived from PBMCs (Fig 3E–3G). Furthermore, the majority of the TIGIT^+^PD-1^+^ HIV-Gag-specific CD8^+^ T cells retained a transitional/intermediate memory (CD45RA^-^CCR7^-^CD27^+^) phenotype (Fig 3H–3J). These results suggest TIGIT may render a large fraction of viral specific CD8^+^ T cells vulnerable to negative regulation.

**Fig 4:**
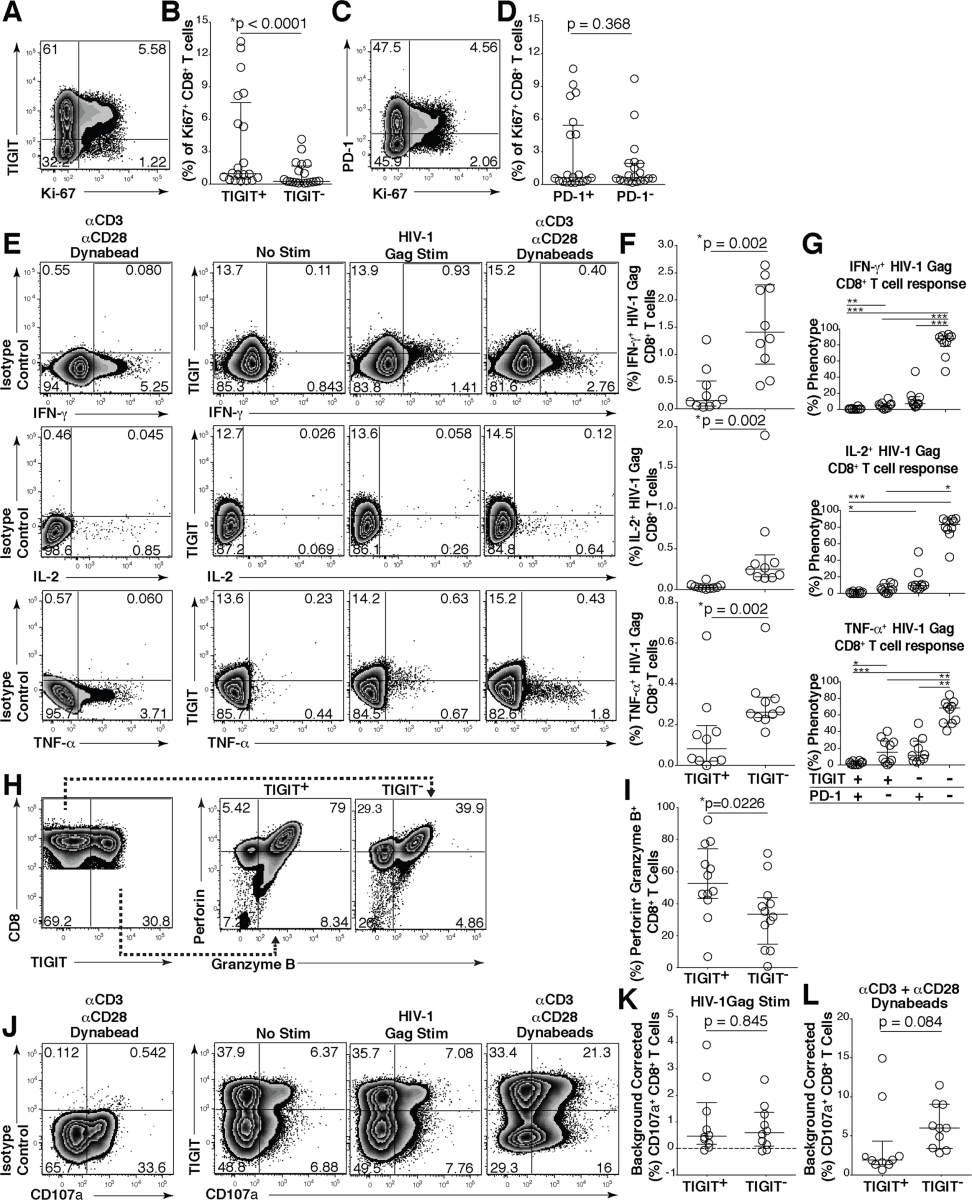
TIGIT expressing CD8^+^ T cells have impaired cytokine responses. Representative flow cytometry plots gated on CD8^+^ T cells showing (A) TIGIT or (C) PD-1 expression against Ki-67 from a chronically HIV-infected individual. Compiled data of Ki-67^+^ CD8^+^ T cell frequency (%) separated into (B) TIGIT^+^ and TIGIT^-^ or (D) PD-1^+^ and PD-1^-^ (*n* = 20). P values were calculated by Wilcoxon matched-pairs signed ranked test. *Ex vivo* PBMCs from chronically HIV-infected individuals were stimulated with HIV Gag peptide pool and assessed for cytokine production. (E) Representative flow cytometry plots gated on CD8^+^ T cells showing TIGIT expression and either IFN-γ, IL-2, or TNF-α content after no stimulation, stimulation with an HIV-1 Gag peptide pool, or a positive control stimulation with anti-CD3 + anti-CD28 Dynabeads. (F) Compiled data of IFN-γ, IL-2, or TNF-α CD8^+^ T cell frequency (%) from TIGIT^+^ or TIGIT^-^ CD8^+^ T cell compartments after HIV-1 Gag peptide pool stimulation (sample group includes; AS *n* = 4, EC *n* = 3, NC *n* = 3). P values were calculated by Wilcoxon matched-pairs signed ranked test. (G) Compiled data of TIGIT and PD-1 expression on HIV-1 Gag responding cells (sample group includes; AS *n* = 4, EC *n* = 3, NC *n* = 3). P values were calculated with repeated-measures one-way ANOVA, followed by Tukey’s multiple comparisons test (*p < 0.05; **p < 0.01; ***p < 0.001). (H) Representative flow cytometry plots of intracellular perforin and granzyme B from CD8^+^ T cells expressing or not expressing TIGIT. (I) Compiled frequency (%) of intracellular perforin^+^granzyme B^+^ content from TIGIT^+^ or TIGIT^-^ CD8^+^ T cell compartments (AS; *n* = 12). P values were calculated by Wilcoxon matched-pairs signed ranked test. (J) Representative flow cytometry plots gated on CD8^+^ T cells showing TIGIT and CD107a expression from TIGIT isotype control, no stimulation, HIV-1 Gag peptide pool, positive control stimulation with anti-CD3 + anti-CD28 Dynabeads. Compiled data of background corrected CD107a after (K) HIV-1 Gag peptide pool (L) anti-CD3 + anti-CD28 Dynabead stimulation in TIGIT^+^ or TIGIT^-^ CD8^+^ T cell compartments (AS; *n* = 10). P values were calculated by Wilcoxon matched-pairs signed ranked test.

Given the high expression of PD-1 among TIGIT^+^ CD8^+^ T cells, we evaluated the functional status of the TIGIT expressing cells. We stained T cells with the nuclear antigen Ki-67, which is associated with cellular proliferation, and observed that TIGIT^+^ cells expressed significantly more Ki-67 than TIGIT^-^ CD8^+^ T cells (Fig 4A and 4B). However, in contrast, Ki-67 expression was equivalently distributed between PD-1^+^ and PD-1^-^ CD8^+^ T cells (Fig 4C and 4D). Using intracellular cytokine staining, in response to stimulation with an overlapping 15mer HIV-1 Gag peptide pool, we observed that TIGIT^+^ CD8^+^ T cells produced significantly less IFN-γ, TNF-α and IL-2 compared to TIGIT^-^ CD8^+^ T cells (Fig 4E and 4F). We observed phenotypically the majority of the HIV specific cytokine responsive CD8^+^ T cells lacked TIGIT and PD-1 dual expression and were minimally represented in the TIGIT^+^PD-1^+^ subset. However, single expressing cells retain some functional responses (Fig 4G).

To directly assess the functionality of the TIGIT^+^PD-1^+^ subset in HIV infected individuals, CD8^+^ T cells expressing TIGIT and/or PD-1 on their surface were sorted to high purities (S3A and S3B Fig), stimulated with or without anti-CD3 + anti-CD28 Dynabeads, and assessed for changes in TIGIT and PD-1 expression and their capacity to secrete 13 different cytokines. We found CD8^+^ T cells lacking TIGIT (TIGIT^-^PD-1^-^ and TIGIT^-^PD-1^+^) robustly upregulated TIGIT upon stimulation (S3B and S3C Fig). Irrespective of PD-1 expression, the TIGIT expressing (TIGIT^+^PD-1^-^ and TIGIT^+^PD-1^+^) cells only marginally increased TIGIT expression (S3B and S3C Fig) upon stimulation. We harvested the supernatants and observed that TIGIT^+^PD-1^+^ cells had the lowest secretion of all cytokines assessed in comparison to the other three subsets (S3D Fig). TIGIT^+^PD-1^-^ cells produced less cytokines than TIGIT^-^PD-1^+^ cells. These data are partially in alignment with results observed in Fig 4G. However, it was notable that IL-10 production was almost exclusively produced by the TIGIT^-^PD-1^+^ cell subset. These data suggest that TIGIT^+^ CD8^+^ T cells, particularly TIGIT^+^PD-1^+^ co-expressing CD8^+^ T cells exhibit distinguishing features of exhausted T cells.

Next, we evaluated the intracellular granular content of TIGIT expressing cells. We observed that TIGIT expressing CD8^+^ T cells contained significantly more perforin and granzyme B compared to non-TIGIT expressing CD8^+^ T cells (Fig 4H and 4I). We observed no difference in the ability of TIGIT^+^ CD8^+^ T cells to degranulate compared to TIGIT^-^ CD8^+^ T cells when stimulated with HIV-1 Gag peptide pool (Fig 4J and 4K). However, upon stimulation with anti-CD3 + anti-CD28 Dynabeads, TIGIT^+^ cells degranulated less than TIGIT^-^ cells (Fig 4J–4L).

### Induction of TIGIT on CD8^+^ T cells during HIV infection

**Fig 5:**
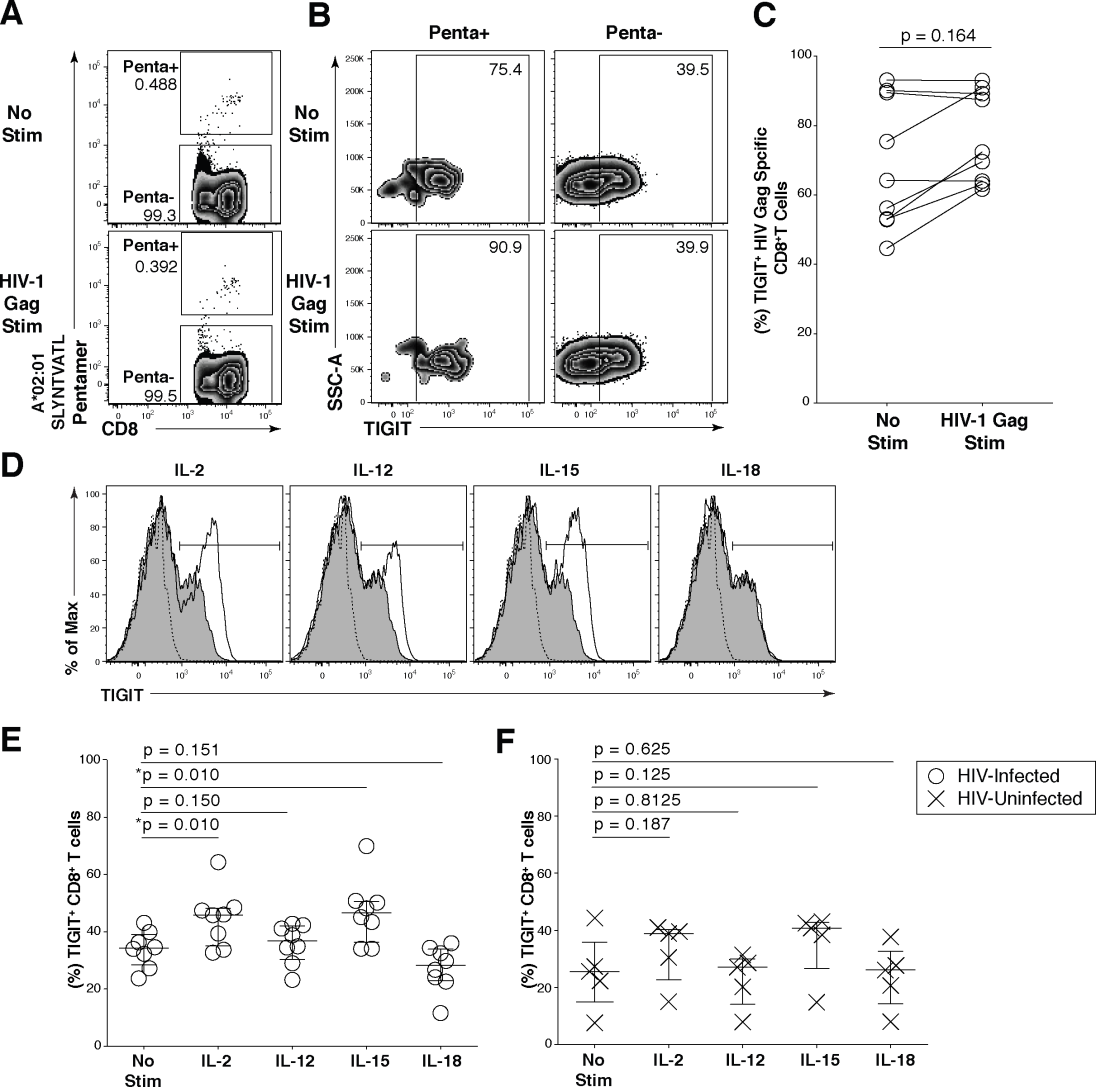
Common γ-chain cytokines regulate TIGIT expression on CD8^+^ T cells. *Ex vivo* PBMCs from chronically HIV-1 infected individuals were stimulated with HIV-1 Gag peptide pool for 12 hours. (A) Representative flow cytometry plot gated on CD8^+^ T cells showing HIV-1 Gag pentamer with no stimulation (top panel) or HIV-1 Gag stimulation (bottom panel). (B) Representative flow cytometry plot of TIGIT expression on Penta+ and Penta- cells with no stimulation or HIV-1 Gag stimulation. (C) Graph shows compiled frequency (%) of TIGIT on Penta+ cells with no stimulation and HIV-1 Gag stimulation (*n* = 9). P values calculated with Wilcoxon matched-pairs signed-rank test. (D) Representative flow cytometry histograms gated on CD8^+^ T cells overlaid with TIGIT expression frequency before and after cytokine stimulation. Dashed line indicates TIGIT isotype control, shaded histogram indicates TIGIT expression with no stimulation, and the solid line indicates TIGIT expression with cytokine stimulation after six days. Compiled data of TIGIT frequency (%) on CD8^+^ T cells (E) HIV-Infected participant (open circle; *n* = 8) (F) HIV-Uninfected participant (X; *n* = 5). P values were calculated with repeated-measures one-way ANOVA, followed by Tukey’s multiple comparisons test.

To explore the regulation of TIGIT expression we stimulated HIV-specific CD8^+^ T cells from chronically HIV-infected individuals with HIV-1 Gag peptides. HIV-1 Gag peptide stimulation did not significantly increase the expression of TIGIT on HIV-specific CD8^+^ T cells, although we did observe an upregulation of TIGIT in a subset of individuals (Fig 5A–5C). Several common gamma-chain (γ-chain) cytokines have been shown to directly upregulate negative checkpoint receptors on CD8^+^ T cells during retroviral infections [35]. To further understand the mechanism driving TIGIT upregulation, we explored the capacity of γ-chain and non-γ-chain cytokines to regulate TIGIT expression (Fig 5D–5F). We found that IL-2 and IL-15 prominently led to a significant increase in TIGIT expression on CD8^+^ T cells from HIV-infected individuals unlike non-γ-chain cytokines IL-12 and IL-18 (Fig 5E). This effect was not evident on CD8^+^ T cells derived from HIV-uninfected participants (Fig 5F). Correspondingly, TIGIT expression on CD4^+^ T cells was upregulated primarily by IL-2 and IL-15 in HIV-infected individuals (S4A Fig). IL-21 stimulation increased TIGIT expression on CD8^+^ T cells, but not CD4^+^ T cells (S4B Fig). These data suggest TIGIT expression may be regulated by a peripheral cytokine milieu dominated by γ-chain cytokines present during HIV infection.

### Effects of anti-TIGIT and anti-PD-L1 mAb blockade on HIV-specific CD8^+^ T cell cytokine and proliferative responses

**Fig 6:**
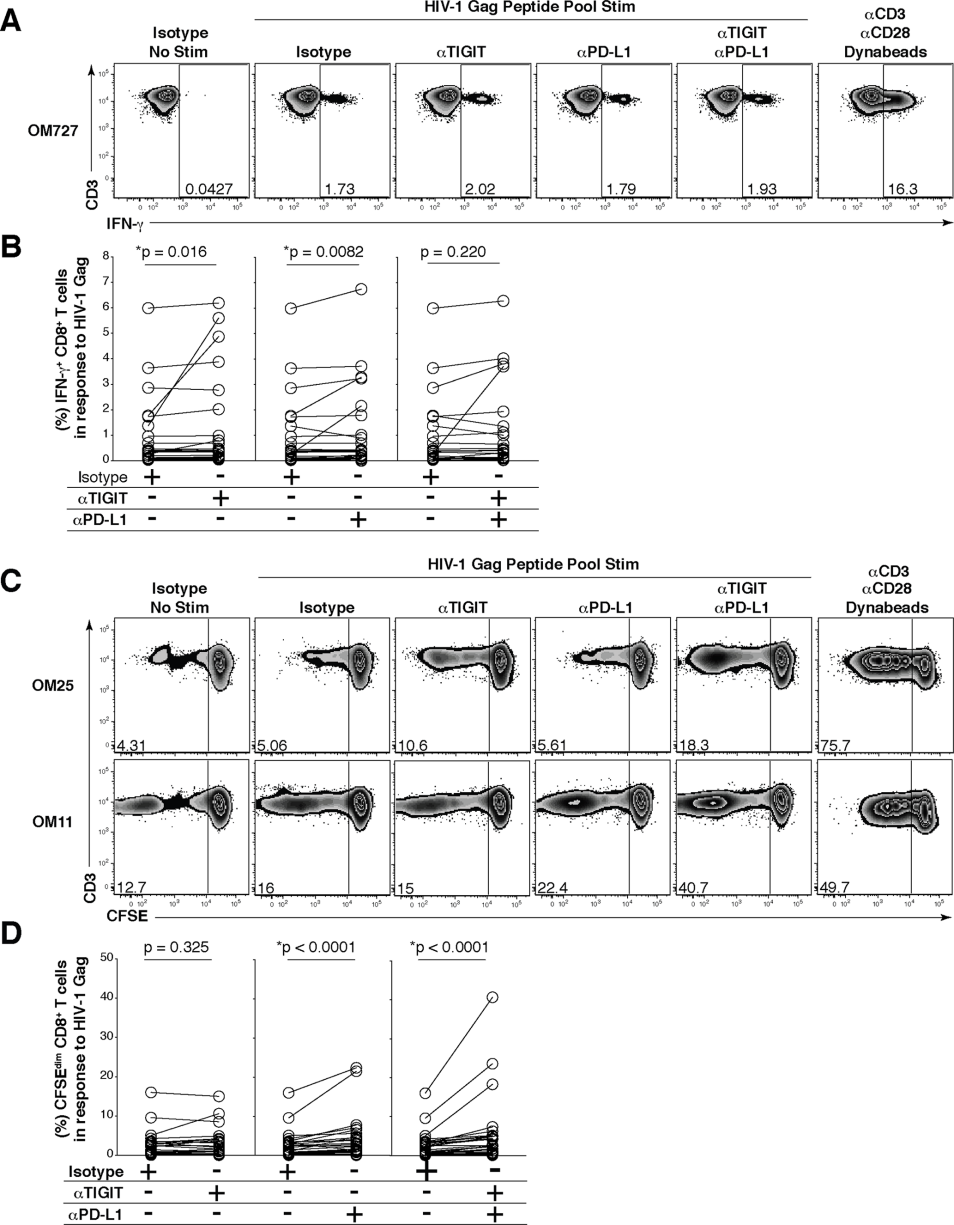
Effect of *in vitro* blockade with anti-TIGIT/anti-PD-L1 mAbs on HIV-specific CD8^+^ T cell responses. *Ex vivo* PBMCs from chronically HIV-infected individuals were stimulated with HIV Gag peptide pool in the presence of mAb blocking antibodies. (A) Representative flow cytometry plots gated on CD8^+^ T cells, showing IFN-γ responses from an HIV-infected individual. No HIV-1 Gag stimulation with an isotype control, HIV-1 Gag stimulation with an isotype control, HIV-1 Gag stimulation with anti-TIGIT, HIV-1 Gag stimulation with anti-PD-L1, HIV-1 Gag stimulation with dual blockade (anti-TIGIT + anti-PD-L1) and a positive control (anti-CD3 + anti-CD28 Dynabeads). (B) Compiled data showing variation in the frequency (%) of IFN-γ in responses to HIV-1 Gag peptide pool with isotype control or mAb blockade; TIGIT blockade (left panel), PD-L1 blockade (middle panel), and dual blockade (right panel) (*n* = 25). P values were calculated by Wilcoxon matched-pairs signed ranked test. (C) Representative flow cytometry plots gated on CD8^+^ T cells from HIV-infected individuals, showing intermediate and high CFSE dilution in response to HIV-1 Gag peptide pool stimulation in the presence of either an isotype control, anti-TIGIT mAb, anti-PD-L1 mAb, a combination of both anti-TIGIT and anti-PD-L1 mAbs or anti-CD3 + anti-CD28 Dynabeads as a positive control. (D) Graphs show compiled data showing variation in the frequency (%) of CFSE^dim^ in responses to HIV-1 Gag peptide pool with either an isotype control or mAb blockade; TIGIT blockade (left panel), PD-L1 blockade (middle panel), and dual blockade (right panel) (*n* = 24). P values were calculated by Wilcoxon matched-pairs signed ranked test.

Since TIGIT and PD-1 are co-expressed, and dual blockade in the mouse model limits *in vivo* LCMV replication [18] and elicits anti-tumor CD8^+^ T cell responses [19], we evaluated the effects of TIGIT and PD-L1 blockade on HIV-Gag-specific CD8^+^ T cells using cells from chronically HIV-infected individuals at various stages of infection (Table 2). To evaluate the *ex vivo* HIV-specific T cell cytokine restoration, we used a modified version of our previously published *in vitro* short-term primary recall blockade assay [8]. Incubation with either anti-TIGIT mAb alone or anti-PD-L1 mAb alone significantly increased IFN-γ production, however dual blockade of both TIGIT and PD-L1 did not enhance IFN-γ responses over anti-TIGIT or anti-PD-L1 alone (Fig 6A and 6B). We also observed that only dual blockade of TIGIT and PD-L1 significantly increased IL-2 production by CD8^+^ T cells (S5A and S5B Fig). Given that virus-specific IL-2 producing CD4^+^ T cells have been associated with disease control in HIV infection we assessed the effects of TIGIT blockade on CD4^+^ T cells [36,37]. Similarly, only dual blockade of TIGIT and PD-L1 significantly increased IL-2 production over the single blockades alone from CD4^+^ T cells (S5C and S5D Fig).

**Table 2:** Description of participants for *in vitro* mAb blockade.

PID	CD4+ T cell Count (cells/mm3)	Viral Load (copies/ml) Log10	On Current cART
AS07-1	543	3.76	No
AS07-2	453	4.08	No
AS09-1	N/A	N/A	N/A
AS09-2	N/A	N/A	N/A
*AS03-1	849	1.79	No
AS08-1	718	1.60	No
*AS11-1	716	1.88	No
*AS12-1	590	1.60	No
*AS14-1	709	1.60	No
AS10-1	784	1.60	Yes
AS10-2	540	1.60	Yes
AS10-3	551	1.60	Yes
AS08-2	843	1.60	Yes
AS10-4	672	1.60	Yes
AS10-5	433	1.60	Yes
AS10-6	1054	4.25	No
AS05-1	475	4.81	No
AS07-3	360	3.87	No
AS08-3	793	1.73	Yes
AS08-4	527	3.72	No
DUKE 012	1819	3.20	No
DUKE 010	1269	1.83	No
OM11	802	4.70	No
OM115	570	3.34	No
OM25	555	4.10	No
OM288	690	1.69	Yes
OM5245	1070	1.60	Yes
OM727	621	5.17	No
OM9	550	1.94	No

AS = SCOPE Cohort

Duke = Duke Cohort

OM = Toronto Cohort

N/A = Not Available

* = Elite Controller

Single blockade of PD-L1 significantly enhanced HIV-specific CD8^+^ T cell proliferation while single blockade of TIGIT did not improve CD8^+^ T cell proliferation (Fig 6C and 6D). When both anti-TIGIT and anti-PD-L1 were combined there was significant increased CD8^+^ T cell proliferation compared to PD-L1 blockade alone (Fig 6C and 6D). Though donor OM115 had the highest baseline levels of TIGIT^+^ CD8^+^ T cells among the group, no significant association was seen between the magnitude of IFN-γ production and proliferation by TIGIT blockade and baseline TIGIT^+^ CD8^+^ T cell expression (r = 0.24, p = 0.257). These data suggest that HIV-specific CD8^+^ T cell proliferation can be markedly improved with simultaneous combination blockade of TIGIT and PD-L1.

### rhTIGIT is elevated on dysfunctional effector CD8^+^ T cells in the SIV rhesus macaque model of HIV/AIDS and is associated with SIV disease progression

**Fig 7:**
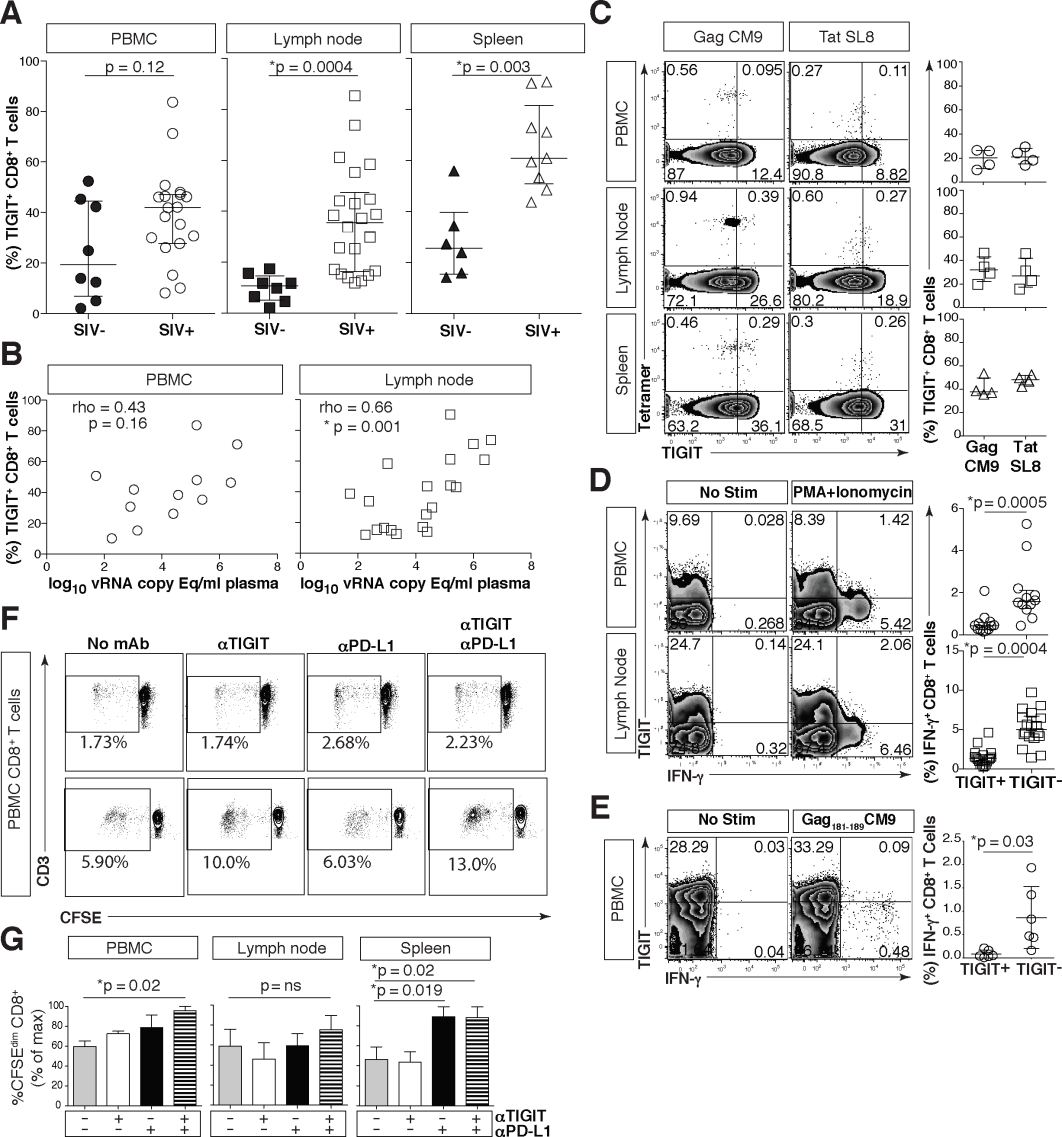
Phenotypic and functional assessment of rhTIGIT expression on CD8^+^ T cells. Cryopreserved rhesus macaque PBMCs were thawed, phenotyped and assessed for function. (A) Graphs show frequency (%) of rhTIGIT^+^CD8^+^ T cells from PBMCs (circle), LNs (square), and spleen (triangle) in SIV-uninfected (filled) and SIV-infected (open) animals (SIV-uninfected PBMCs, *n* = 8; SIV-infected PBMCs, *n* = 19; SIV-uninfected LNs, *n* = 8; SIV-infected LNs, *n* = 22; SIV-uninfected spleen, *n* = 6; SIV infected spleen, *n* = 9). P values were calculated with Mann-Whitney U tests. (B) Graphs show correlation of frequency (%) of rhTIGIT^+^ CD8^+^ T cells in PBMCs (circle) and lymph nodes (square) from SIV-infected animal against plasma SIV viral load log_10_ vRNA copy Eq/ml (PBMCs, *n* = 12; LNs, *n* = 20). vRNA copy Eq, viral RNA copy equivalents. Spearman’s rho tests were performed for correlations. (C) Representative flow cytometry plots of tetramer stains for Mamu-A*01 restricted SIV-Gag CM9 and SIV-Tat SL8 specific CD8^+^ T cells from PBMC, LNs, and spleen, in a representative Mamu-A*01 animal with full cART suppression. Compiled data of rhTIGIT expression frequency (%) on tetramer specific CD8^+^ T cells (*n* = 4) from PBMCs (circle), LNs (square), and spleen (triangle) from Mamu-A*01^+^ macaques with full cART suppression. (D) Representative flow cytometry plots of PBMCs (*n* = 12) or lymph nodes (*n* = 18) stimulated without or with PMA + Ionomycin. Graphs show frequency (%) of IFN-γ from CD8^+^ T cells expressing TIGIT or not expressing TIGIT. P values were calculated by Wilcoxon matched-pairs signed ranked test. (E) Representative flow cytometry plots of PBMCs from a Mamu-A*01+ macaque stimulated without or with SIV-Gag_181-189_ CM9 peptide. Graph shows frequency (%) of IFN-γ from SIV-Gag_181-189_ CM9-specific CD8^+^ T cells expressing TIGIT or not expressing TIGIT from Mamu-A*01^+^ macaques (*n* = 6). P values were calculated by Wilcoxon matched-pairs signed ranked test. (F) Representative flow cytometry plots of CD8^+^ T cells from two separate SIV-infected macaques showing CD8^+^ T cell CFSE dilution in response to AT2-inactivated SIV with either no antibody, anti-TIGIT, anti-PD-L1 or dual blockade (anti-TIGIT + anti-PD-L1). (G) Graphs show compiled data of CD8^+^ T cell CFSE dilution from PBMC (left panel), Lymph node (middle panel), or Spleen (right panel) as percent of max (*n* = 4). P values were calculated by Wilcoxon matched-pairs signed ranked test.

To explore the role of TIGIT in the rhesus macaque model of HIV/AIDS we cloned rhesus TIGIT (rhTIGIT) (GenBank: KR534505) and observed that it shares 88.11% sequence homology with human TIGIT (S6A Fig). We reasoned that rhTIGIT expression and function would mimic our human HIV studies and be replicable in the SIV-infected rhesus macaque model of HIV/AIDS. RhTIGIT expression was significantly increased on CD8^+^ T cells derived from the lymph node (LN) (38.6%) and splenic (60.9%) compartments when compared to SIV-uninfected macaques (LN 10.82% and spleen 25.55%), but not in PBMCs (Figs 7A and S6B). Similar to what we observed in HIV-infected participants, the frequency of rhTIGIT^+^ CD8^+^ T cells from PBMC did not correlate with plasma SIV viral load. However, we did observe a significant correlation with the frequencies of rhTIGIT^+^ CD8^+^ T cells in LN and viral load (Fig 7B).

As observed in human HIV infection, rhTIGIT expression was more prominently expressed in SIV infection on effector memory (EM, CD28^-^CD95^+^), and central memory (CM, CD28^+^CD95^+^) CD8^+^ T cells when compared to naïve (N, CD28^+^CD95^-^) CD8^+^ T cells from PBMCs, LN and from the spleen (S6C Fig). In the tissues, it was notable that TIGIT expression was highest on the central memory CD8^+^ T cells when compared to PBMCs (S6C Fig). As in HIV infection, stimulation with γ-chain cytokines such as IL-2 and IL-15 upregulated rhTIGIT on CD8^+^ T cells from SIV-infected animals (S6D Fig).

rhTIGIT was also expressed on ~40% of SIV Gag or Tat tetramer specific CD8^+^ T cells derived from PBMCs or secondary lymphoid tissues, even in animals with full cART suppression of peripheral SIV viremia (Fig 7C). This was more prominently found in the tissues of SIV-infected animals where higher frequency of SIV-specific CD8^+^ T cells co-expressed both rhTIGIT and rhesus macaque PD-1 (rhPD-1) (S6E–S6H Fig).

While the levels of Ki-67 expression did not differ between rhTIGIT^+^ and rhTIGIT^-^ CD8^+^ T cells from SIV-infected rhesus macaques (S7A–S7D Fig), CD8^+^ T cells lacking rhTIGIT from PBMC produced significantly more IFN-γ compared to rhTIGIT^+^ CD8^+^ T cells when stimulated with either phorbol 12-mysistate 13-acetate (PMA) + ionomycin or SIV Gag_181-189_ CM9 peptide (Fig 7D and 7E).

Given the similarities of rhTIGIT and human TIGIT, we evaluated TIGIT and PD-L1 blockade on SIV peptide stimulated CD8^+^ T cell responses. We found that dual blockade of rhPD-L1 and rhTIGIT enhanced SIV-specific CD8^+^ T cell proliferation in PBMCs and spleen while single blockade of rhPD-L1 enhanced SIV-specific proliferation in the spleen (Fig 7F and 7G). Taken together, rhTIGIT pathway is active in the rhesus macaque model of HIV/AIDS and partially mimics human TIGIT expression and function during HIV infection.

## Discussion

In this report we profiled TIGIT expression on T cells in HIV-infected participants with various degrees of viral control and in SIV-infected rhesus macaques. We (1) unveil a role for TIGIT^+^ CD8^+^ T cells in HIV disease progression and demonstrate its relation to T cell exhaustion, (2) observe that TIGIT appears to associate with the cellular viral reservoir in CD4^+^ T cells, (3) we found that co-blockade of TIGIT and PD-L1 lead to a greater restoration of T cell function compared with a single blockade, and (4) by successfully cloning rhTIGIT (GenBank: KR534505) we reveal the similarities in expression and function of rhTIGIT on T cells in the non-human primate model of HIV/AIDS. Our findings reveal a novel inhibitory pathway involved in the suppression of T cell responses during chronic viral infection, the blockade of which may contribute to the reversal of T cell dysfunction in the control or elimination of HIV infection.

While TIGIT levels on CD8^+^ T cells tracked disease progression (depletion of CD4 T cells and T cell activation), this was not evident across the various HIV-infected groups. There was a significant difference in TIGIT^+^ memory CD8^+^ T cells in acute infection compared to the uninfected group, but not in the global CD8^+^ T cell population. This suggests there may be a gradient, with an increase in global TIGIT^+^ CD8^+^ T cells in acute infection that becomes greater over time, which is distributed among the differentiated CD8^+^ T cell populations and may differ when compared to other negative checkpoint receptors which are found elevated during the early stages of HIV infection [4,38,39].

TIGIT induction appears to be driven by polyclonal TCR stimulation and this is a common feature among immune checkpoint receptors [38,40]. We observed HIV-1-Gag-SL9-specific CD8^+^ T cells did not increase TIGIT expression after HIV-1 Gag peptide stimulation as a group, however a subset of individuals with moderate levels of TIGIT increased expression after stimulation, and individuals that expressed high levels of TIGIT retained expression after stimulation. TIGIT remained elevated despite antigen in cART-suppressed individuals; previous studies have also shown that common γ-chain cytokines maintain the ability to regulate immune checkpoint receptor expression in the absence of antigenic stimulation [35,41]. Our studies align with these previous observations and suggest that a cytokine milieu conducive for the maintenance of an exhausted T cell profile persists in HIV and SIV infection even during viral suppression.

TIGIT expression was found to be associated with T cell activation principally among EC who represent a small population of HIV-infected individuals able to spontaneously suppress their viral load (<50 copies/ml) in the absence of cART for prolonged periods of time [42]. However, over time a subset of EC will lose virologic control and develop viremia and CD4^+^ T cell loss [43,44]. In addition, EC maintain elevated levels of T cell activation despite viral control [45,46]. High TIGIT expression may reflect ongoing immune activation in the EC population. The institution of cART in those EC has led to a reduction in immune activation [47,48]. Given our finding, in addition to cART, some EC may benefit from TIGIT blockade to alleviate the persistent T cell immune activation thereby reducing the risk of adverse non-AIDS events that have been documented to occur in this population, however such strategies need to be considered carefully given the risk of autoimmunity as described in anti-PD-1 clinical trials in the oncology field [49,50].

Viral clearance of the chronic strain of LCMV (clone 13) in mice by combined blockade of TIGIT and PD-L1 provided the first evidence of the advantages of targeting these two pathways [18]. In addition, targeting TIGIT and PD-L1 on CD8^+^ tumor infiltrating lymphocytes in patients with advanced melanoma synergistically improves potent anti-tumor responses [19]. Here we extend these finding to human and simian retroviral infections. This was significant given the expansion of dual expressing TIGIT and PD-1 CD8^+^ T cells in HIV infection despite pharmacological or immunological viral suppression. Our data shows the presence of TIGIT and PD-1 dual expressing HIV and SIV-specific CD8^+^ T cells and co-blockade of TIGIT and PD-L1 better enhanced proliferation of HIV and SIV-specific CD8^+^ T cell responses compared to single blockade. Although we see a significant increase among all HIV-infected individuals, it was evident that subsets of weak-responders exist and appear heterogeneous irrespective of stage of infection, viral load levels or viral suppression. Indeed, combinational blockade of CTLA-4 and PD-1 revealed a subset of weak-responders to anti-tumor activity. Different or expanded combinations of immune checkpoint blockers with anti-TIGIT may need to be considered in the arsenal to improve anti-viral T cell immunity in all individuals.

Persistence of the cellular latent HIV reservoir has been a major barrier to the eradication of HIV [51]. One proposed strategy is to ‘Shock’ the latently infected cells to flush out virus with latency reversal agents (LRAs) [52–54]. The development of the ‘Shock’ strategies is advancing at a rapid pace with *in vivo* studies yielding activity in reactivating of latent virus. However, the ‘Kill’ component is less well developed. Shan and colleagues demonstrate that after reactivation of latent virus from CD4^+^ T cells, CD8^+^ T cells’ activity had the capacity to kill latently infected CD4^+^ T cells if appropriate pre-stimulation of HIV peptide and IL-2 was provided an *in vitro* latency assay using Bcl-2 as a survival signal to prolong the longevity of the latent CD4^+^ T cells [55]. Furthermore, recent studies show that HIV-infected individuals on cART retain broad HIV-specific cytotoxic T-lymphocyte responses that are able to target the mutated latent virus [56]. Blocking immune checkpoint pathways such as TIGIT and PD-1/PD-L1 can be harnessed to boost HIV-specific CD8^+^ T cells responses given that these pathways persist in the setting of viral suppression. Furthermore, given our findings showing the relationship with TIGIT expression on CD4^+^ T cells and the total cell associated HIV DNA, it remains unclear what role TIGIT may play in the establishment of the reservoir in CD4^+^ T cells, however it is likely related to the capacity of TIGIT’s ability control T cell activation or proliferation.

Our study provides a novel role for TIGIT during HIV disease pathogenesis and our demonstration of a role of rhTIGIT in the non-human primate model of HIV/AIDS provides a platform to investigate our understanding of the complex networks of co-inhibition that can be tailored to each individual or viral infection. Improving CD8^+^ T cell functions may further aid in the ‘Shock and Kill’ approaches being considered to eliminate latent virus and improve T cell mediated vaccine responses to prevent or limit infection [57].

## Materials and Methods

### Study participants

We recruited participants from the following cohorts: University of California, San Francisco (UCSF) SCOPE and OPTIONS cohorts [8] the Hawaii HIV-1 (HHC) cohort, the Toronto-based cohort CIRC (Maple Leaf Clinic and St. Michael’s Hospital, Toronto, Canada) [58] and the Duke Human Vaccine Institute (DHVI) tissue repository. SCOPE specimens (*n* = 80) were selected from the following groups: untreated non-controllers (*n* = 20) (participants who had never been treated with antiretroviral agents or who had been off therapy for at least six months), treated virologic controllers (participants who had an undetectable plasma HIV-1 RNA level for the previous six months while on cART) (*n* = 20), spontaneous “elite” virologic controllers (participants who are untreated and who have at least three documented plasma HIV-1 RNA levels <2,000 copies/ml over at least a 12-month period) (*n* = 20), Some of these participants had persistent plasma HIV-1 RNA levels <75 copies/ml) and HIV-infected “cART initiators” (*n* = 20) who meet strict selection criteria and well documented persistent viral suppression for over 1.5 years. Participants with acute HIV infection (*n* = 24) were obtained for the OPTIONS cohort of primary HIV infection [8] and age-matched HIV-uninfected (*n* = 20) (Table 1) and chronically infected virally suppressed leukapheresesed individuals were obtained from the HAHC cohort [59]. Additional participants with chronic infection at various stages of infection were obtained from participants with various levels of viral control from the Toronto-based cohort CIRC cohort, HHC, and DHVI.

### Ethics statement

All persons gave written informed consent to participate in the study and approval for the study was obtained from the University of Hawaii Committee of Human Subjects. Samples were obtained from Indian rhesus macaques (*Macaca mulatta*) housed at the Oregon National Primate Research Center (ONPRC), which were SIV infected for other ongoing, unrelated studies. Oregon Health and Science University (OHSU) Institutional Animal Care and Use Committee (IACUC) Protocol #: 0989. The OHSU Institutional Animal Care and Use Committee reviewed and approved all study protocols. All macaques in this study were managed according to the ONPRC animal husbandry program, which aims at providing consistent and excellent care to nonhuman primates. This program is based on the laws, regulations, and guidelines set forth by the United States Department of Agriculture (e.g., the Animal Welfare Act and its regulations, and the Animal Care Policy Manual), Institute for Laboratory Animal Research (e.g., Guide for the Care and Use of Laboratory Animals, 8^th^ edition), Public Health Service, National Research Council, Centers for Disease Control, and the Association for Assessment and Accreditation of Laboratory Animal Care (AAALAC) International. The nutritional plan utilized by the ONPRC is based on National Research Council recommendations and supplemented with a variety of fruits, vegetables, and other edible objects as part of the environmental enrichment program established by the Behavioral Management Unit. Paired/grouped animals exhibiting incompatible behaviors were reported to the Behavioral Management staff and managed accordingly. All efforts were made to minimize suffering through the use of minimally invasive procedures, anesthetics, and analgesics when appropriate. Animals were painlessly euthanized with sodium pentobarbital and euthanasia was assured by exsanguination and bilateral pneumothorax, consistent with the recommendations of the American Veterinary Medical Guidelines on Euthanasia (2013)

### Quantifying cellular HIV-1 DNA and RNA

Cryopreserved PBMCs were rapidly thawed and enriched for CD4^+^ T cells to high purities with an EasySep Human CD4^+^ T cell enrichment kit (Stemcell Technologies, Vancouver, British Columbia, Canada). Cellular RNA and DNA from PBMC T-cell subsets cells were purified using the AllPrep DNA/RNA kit (Qiagen, Ventura CA) as specified by the manufacturer, quantified using a Nanodrop (ND-1000) spectrophotometer and normalized to cell equivalents by qPCR using human genomic TERT for DNA and GAPDH or RPLP0 expression for RNA (Life Technologies, Grand Island NY). Total cellular HIV-1 DNA (integrated and unintegrated) and RNA (unspliced and multiply spliced) was quantified with a qPCR TaqMan assay using LTR-specific primers F522-43 (5’ GCC TCA ATA AAG CTT GCC TTG A 3’; HXB2 522–543) and R626-43 (5’ GGG CGC CAC TGC TAG AGA 3’; 626–643) coupled with a FAM-BQ probe (5’ CCA GAG TCA CAC AAC AGA CGG GCA CA 3) [60] on a StepOne Plus Real-time PCR System (Applied Biosystems Inc, Foster City CA). Cell associated HIV-1 DNA copy number was determined using a reaction volume of 20 μl with 10 μl of 2x TaqMan Universal Master Mix II including UNG (Life technologies), 4 pmol of each primer, 4 pmol of probe, and 5 μl of DNA. Cycling conditions were 50°C for 2 min, 95°C for 10 min, then 60 cycles of 95°C for 15s and 59°C for 1 min. Cell associated HIV-1 RNA copy number was determined in a reaction volume of 20 μl with 10 μl of 2x TaqMan RNA to Ct 1 Step kit (Life Technologies), 4 pmol of each primer, 4pmol of probe, 0.5 μl reverse transcriptase, and 5μl of RNA under identical cycling conditions. For HIV-1 DNA measurements, external quantitation standards were prepared from pNL4-3 in a background of HIV-1 negative human cellular DNA, calibrated to the Virology Quality Assurance (VQA, NIH Division of AIDS) cellular DNA quantitation standards. For HIV RNA measurements, external quantitation standards were prepared from full length NL4-3 virion RNA followed by copy number determination using the Abbott RealTime assay (Abbott Diagnostics, Des Plains Ill) and calibrated to VQA HIV-1 RNA standards. Patient specimens were assayed with up to 800 ng total cellular RNA or DNA in replicate reaction wells and copy number determined by extrapolation against a 7-point standard curve (1–10,000 cps) performed in triplicate.

### Antibodies and flow cytometric analysis

Cryopreserved PBMC were rapidly thawed in warm 10% cRPMI (RPMI 1640 medium; (Hyclone, Logan, Utah) supplemented with 10% fetal bovine serum (FBS) (Hyclone), 1% penicillin-streptomycin (Hyclone), 10 mM HEPES (Hyclone), 2 mM L-glutamine (Hyclone), and 10 μg/ml DNase I (Sigma-Aldrich, Dorset, United Kingdom), washed with PBS + 2% FBS (Hyclone) (complete RPMI). Cells were stained for viability with an aqua amine reactive dye (AARD; Invitrogen, Carlsbad, California), then incubated with panels of conjugated anti-human monoclonal antibodies (mAbs) The following directly conjugated mAbs used in this study were obtained from BD biosciences (San Jose, California): PE-Cy5-conjugated anti-CD38 (Clone: HIT2), V450-conjugated anti-CD45RA (HI100), FITC-conjugated anti-CD45RA (HI100), PerCP-Cy5.5-conjugated anti-CD27 (M-T271), Alexa Flour 700-conjugated anti-CD4 (RPA-T4), FITC-conjugated anti-HLA-DR (G46-6), APCH-7-conjugated anti-CD8 (SK1), FITC-conjugated anti-CD57 (NK-1), APC-conjugated CD107α (H4A3). mAb obtained from Beckman Coulter (Fullerton, California) ECD-conjugated anti-CD3 (UCHT1). mAbs obtained from eBioscience (San Diego, California) PE-Cy7-conjugated anti-CD28 (CD28.2), PerCP-eFluor 710-conjugated anti-TIGIT (MBSA43), PE-conjugated anti-TIGIT (MBSA43), Mouse IgG1 Kappa isotype control PerCP-eFluor 710 (P3.6.2.8.1), mouse IgG1 K isotype control PE (P3.6.2.8.1). mAbs obtained from Biolegend (San Diego, California) Brilliant Violet 605-conjugated anti-CCR7 (G043H7), APC-conjugated anti-PD-1 (EH12.2H7), mouse IgG1 Kappa isotype control PE (MOPC-21). Qdot 605-conjugated anti-CD8 (3B5) was obtained from Invitrogen (Carlsbad, California). In some experiments cells were fixed with 1X Lyse buffer (BD Biosciences) followed by 1X BD FACS Permeabilizing solution 2 (BD Biosciences) and stained with FITC-conjugated Ki-67 (35/Ki-67), FITC-conjugated interferon gamma (IFN-γ) (25723.11), Alexa 700-conjugated Granzyme B (GB11), PE-conjugated perforin (B-D48) (abcam, Cambridge, Massachusetts). All cells were washed with PBS + 2% FBS and then fixed in 1% paraformaldehyde (PFA, Electron Microscopy Sciences, Hatfield, Pennsylvania) before acquiring (within 18 hours) on a custom four laser LSRFortessa flow cytometer (BD Biosciences). Between 100,000 to 500,000 lymphocyte events were collected for each sample. Isotype controls or fluorescence minus one (FMO) samples were prepared to facilitate gating. Anti-mouse or anti-rat IgG-coated beads (BD Biosciences) were individually stained with each fluorochrome-conjugated antibody and used for software-based compensation. Data were analyzed using Flowjo Software version 9.5 (Treestar, Ashland, Oregon).

### Cell sorting

Cryopreserved PBMCs were rapidly thawed and enriched for CD8^+^ T cells to high purities with an EasySep Human CD8^+^ T cell negative selection enrichment kit (Stemcell). Cells were surface stained with the following combination of mAbs: BV711-conjugated anti-CD3, Alexa 700-conjugated anti-CD4 (BD Biosciences), PerCP-eFluor 710-conjugated anti-TIGIT (eBioscience), Qdot605-conjugated anti-CD8, APC-conjugated anti-PD-1 (Invitrogen). Cells were sorted on a BD FACS ARIA and checked for purity. Gating was facilitated by isotype controls.

### Multiplex cytokine assay

The four-sorted populations (TIGT^+^PD-1^+^, TIGIT^+^PD-1^-^, TIGIT^-^PD-1^+^, TIGIT^-^PD-1^-^) were seeded at 100,000 cells per well in a 96 well culture plate with 200 μl of 10% cRPMI. Sorted cells were stimulated with anti-CD3 + anti-CD28 Dynabeads (Life Technologies) for 48 hours in an incubator at 37°C with 5% CO_2_, supernatants were harvested from the cultures and processed according to recommended manufacture procedure with a Milliplex MAP Human High Sensitivity T cell Panel (EMD Millipore, Billerica, Massachusetts) for GM-CSF, TNF-α, IL-13, IL-12 (p70), IL-10, IL-8, IL-7, IL-6, IL-5, IL-4, IL-2, IL-1β, IFN-γ. Samples were acquired on a Luminex 200 (EMD Milipore). Samples were run in duplicate. The intra-assay CV% for the conditions of each cytokines were <10%.

### Pentamer analysis

We used the following Biotin labeled pentamers: A*02:01 SLYNTVATL HIV-1-Gag, A*02:01 ILKEPVHGV HIV-1-Pol, B*07:02 IPRRIRQGL HIV-1-Env, B*07:02 TPGPGVRYPL HIV-1-Nef, and A*02:01 NLVPMVATV CMV-pp65-NV9. All pentamers were obtained from Proimmune Ltd, Oxford, UK. Using protocol outlined previously [8] and stained with antibodies against CD3, CD8, TIGIT, PD-1, TIGIT isotype control or PD-1 isotype control and acquired on the flow cytometer as above. In some experiments PBMCs were stimulated with HIV-1 Gag Peptide pool and evaluated for pentamer phenotype

### anti-TIGIT and anti-PD-L1 monoclonal antibodies

The TIGIT antibody clones 11G11 and 23G8 were generated in HuMab mice [61,62] immunized with a TIGIT-Fc fusion protein and selected based on their high affinity for TIGIT and ability to block TIGIT/PVR interaction. Clone 11G11 is a fully human IgG1 antibody that was engineered to contain a well-characterized set of mutation in the Fc that eliminate FcR interaction [63]. Clone 23G8 is a fully human IgG2 antibody that cross-reacts with macaque TIGIT. The anti-human PD-L1 antibody, clone 12A4, is a fully human IgG4 (S228P) that was generated in HuMab mice immunized with PD-L1-Fc. This antibody was selected based on its ability to block the binding of PD-L1 to both PD-1 and CD80. 12A4 cross-reacts with macaque PD-L1.

### Peptides and polyclonal stimulation

123 Overlapping ~15mer HIV-1 clade B gag peptides obtained from the National Institute of Health AIDS Reagent Program. Stimulations were performed with a final concentration of 10 μg/ml peptide. T cell activator (anti-CD3 + anti-CD28 mAb Dynabeads) (Life Technologies) followed recommended manufacture procedure.

### Functional assays

In the intracellular cytokine stimulation assay studies, thawed cryopreserved PBMCs were stimulated for 12 hours in an incubator at 37°C with 5% CO_2_ with 5 μg/ml brefeldin A and 5 μg/ml monensin (Sigma-Aldrich) culture media, DMSO alone, pooled HIV-1 Gag peptides, or anti-CD3/CD28 dynabeads (Life Technologies) in the presence or absence of purified isotype IgG control, anti-TIGIT and/or anti-PD-L1 mAbs. After stimulation, the cells were washed and stained for viability with AARD and cultured with surface phenotype panel against CD8, TIGIT or an isotype control antibody, followed by intracellular staining of CD3 and IFN-γ and acquisition on the flow cytometer as above. For the proliferation assay, thawed PMBCs were washed two times and resuspended in PBS supplemented with 0.01% BSA at a concentration of one million cells per milliliter. Cells were labeled with 1mM Carboxyfluorescein succinimidyl ester (CFSE) violet trace (Invitrogen) using protocols previously described [8]. Cells were stimulated for seven days with DMSO alone, pooled HIV-1 gag peptides, or anti-CD3/CD28 Dynabeads in the presence or absence of purified isotype IgG control, anti-TIGIT or anti-PD-L1 at 37°C with 5% CO_2_. At the end of the stimulation, cells were washed and stained for viability with AARD and cultured with surface phenotype panel against CD3, CD8, TIGIT or an isotype control antibody and acquired on the flow cytometer as above.

### *In vitro* cytokine stimulation

PBMCs were thawed and one million cells were plated per stimulation condition. Stimulation conditions included media alone, 25 ng/ml IL-2 (Roche), 50 IU/ml of IL-12 (MBL international, Woburn, Massachusetts), 50 IU/ml of IL-18 (MBL international) and 25 ng/ml IL-15 (R&D Systems, Minneapolis, Minnesota). Cells were stimulated for six days in an incubator at 37°C with 5% CO_2_. After stimulation, the cells were washed and stained for viability with AARD cultured with surface phenotype panel against CD3, CD4, CD8, TIGIT or isotype control antibody and acquired on the flow cytometer as above.

### T cell function in vitro antibody blockade studies

HIV-infected cryopreserved PBMC from individuals identified in Table 2 were stimulated for 12 hours in an incubator at 37°C with 5% CO_2_. Stimulation conditions contained culture media, DMSO alone or pooled HIV-1 gag peptides, in the presence of brefeldin A (Sigma-Aldrich), monensin (Sigma-Aldrich), anti-TIGIT mAb anti-PD-L1 mAb or mouse IgG1 isotype control. After stimulation, cells were washed and stained for cellular viability with AARD and conjugated antibodies against CD8 and CD4, followed by intracellular staining of CD3 and IFN-γ and acquired on a flow cytometer as described above.

### Statistical analysis

The repeated-measures, one-way ANOVA followed by Tukey’s multiple comparison, Wilcoxon matched-pairs signed ranked test was used for paired tests and the Spearman’s rho test was used for correlation analyses. Measures of central tendency are expressed as medians and interquartile ranges (IQRs; given in the form 25th percentile, 75th percentile). Statistical analyses were conducted using Prism Graphpad release 5.0d (Graphpad Software, San Diego, California) or SPSS 22.0 (IBM, Armonk, New York) and the statistical significance of the findings was set at a *p*-value of less than 0.05.

### Accession numbers

Indian rhesus macaque (Macaca mulatta) TIGIT (rhTIGIT): GenBank KR534505.

## Supporting Information

S1 Text Supplemental methods. (DOCX)

S1 Fig Gating strategy of TIGIT surface expression in HIV infection and associations with HIV clinical parameters. (A) Representative flow cytometry plots showing gating scheme to isolate CD8^+^ and CD4^+^ T cells. Gated on singlets, excluded dead cells, gated on lymphocytes, gated on CD3^+^ T cells, and gated on expression of CD8 or CD4. (B) Representative histograms of TIGIT Isotype, TIGIT FMO and TIGIT expression on CD8^+^ or CD4^+^ T cells (HIV-Infected thin solid line, HIV-Uninfected dashed line, TIGIT isotype control shaded, and TIGIT FMO thick solid line). Graphs show the association of the frequency (%) of (C) TIGIT^+^ CD8^+^ or (D) TIGIT^+^ CD4^+^ T cells against clinical CD4 count for cART suppressed (left panel, open triangle, *n* = 20) and elite controllers (right panel, open squares, *n* = 20). Graphs show the association of the frequency (%) of (E) TIGIT^+^ CD8^+^ or (F) TIGIT^+^ CD4^+^ T cells against T cell activation (% CD38^+^HLA-DR^+^) for cART suppressed (left panel, open triangle, *n* = 20) and non-controllers (right panel, open circles, *n* = 20). Graphs show the association of the frequency (%) of (G) TIGIT^+^ CD8^+^ or (I) TIGIT^+^ CD4^+^ T cells against viral load log_10_ (copies/ml) for non-controllers (open circles, *n* = 20). Graphs show the association of the frequency (%) of (H) TIGIT^+^ CD8^+^ or (J) TIGIT^+^ CD4^+^ T cells against copies of cell associated HIV RNA per million CD4^+^ T cells for L-AS (inverted open triangles, *n* = 19). Spearman’s rho tests were performed for correlations.
(TIF)

S2 Fig Phenotypic assessment of TIGIT expression on differentiated CD8^+^ T cell subsets. (A) Graph shows compiled frequency (%) of TIGIT expression on CD8^+^ T cells subsets grouped by disease category. HIV-Uninfected (X; *n* = 20), acute infected (AI; open diamond; *n* = 24), cART suppressed (AS; open triangle; *n* = 20), elite controller (EC; open square; *n* = 20), and non-controllers (NC; open circle; *n* = 20). Repeated-measures one-way ANOVA, followed by Tukey’s multiple comparisons test were used for comparison (*p < 0.05; **p < 0.01; ***p < 0.001). Cryopreserved PBMCs from chronically HIV-infected individuals were phenotyped for TIGIT expression on CD8^+^ T cell subsets. (B) Representative flow cytometry plots showing gating scheme to isolate CD8^+^ T cell subsets. Live lymphocytes gated for CD8^+^ T cells, subset into CD45RA^+^ and CD45RA^-^, further stratified by expression of CCR7 and CD27. (C) Representative flow cytometry plots showing CD28 expression on CD8^+^ T cell subsets. (D) Representative flow cytometry plots showing TIGIT expression on CD8^+^ T cell subsets. (E) Graph shows compiled frequency (%) of TIGIT expression on CD8^+^ T cell subsets (*n* = 20).
(TIF)

S3 Fig Cytokine profile of TIGIT and PD-1 expressing CD8^+^ T cells. *Ex vivo* CD8^+^ T cells from chronically HIV-infected individuals were FACS sorted into populations according to their expression of TIGIT and PD-1. (A) Representative flow cytometry plot of TIGIT and PD-1 expression PRE-SORT. Gating was facilitated by isotype controls for TIGIT and PD-1. (B) Representative flow cytometry plots of CD8^+^ T cells sorted into TIGIT^+^PD-1^+^, TIGIT^+^PD-1^-^, TIGIT^-^PD-1^+^, and TIGIT^-^PD-1^-^. No stimulation (left panel) and stimulated with anti-CD3 + anti-CD28 Dyanbeads for 48 hours (right panel). (C) Graphs show compiled data of phenotypes of sorted populations with no stimulation (open box) and anti-CD3 + anti-CD28 Dyanbeads (filled box) (*n* = 2). Supernatants were harvested and cytokine production was assessed 48 hours post anti-CD3 + anti-CD28 stimulation by high sensitivity multiplex bead array. (D) Graphs show concentrations of cytokines produced from sorted populations.
(TIF)

S4 Fig Cytokine regulation of TIGIT expression. (A) Compiled data of HIV-Infected individuals (open circle; *n* = 8) TIGIT expression frequency (%) on CD4^+^ T cells with or without cytokine stimulation for six days. P values were calculated with repeated-measures one-way ANOVA, followed by Tukey’s multiple comparisons test (*p < 0.05). (B) Compiled data of HIV-Infected individuals (open circle; *n* = 6) TIGIT expression frequency (%) on CD8^+^ T cells (right panel) and CD4^+^ T cells (left panel) after six days of IL-21 stimulation (*n* = 6). P values were calculated by Wilcoxon matched-pairs signed ranked test.
(TIF)

S5 Fig Effect of *in vitro* blockade with anti-TIGIT/anti-PD-L1 mAbs on HIV-specific CD8^+^ T cell IL-2 responses. *Ex vivo* PBMCs from chronically HIV-infected individuals were stimulated with HIV Gag peptide pool in the presence of mAb blocking antibodies. Representative flow cytometry plots gated on (A) CD8^+^ or (C) CD4^+^ T cells, showing IL-2 responses from an HIV-infected individual. No HIV-1 Gag stimulation with an isotype control, HIV-1 Gag stimulation with an isotype control, HIV-1 Gag stimulation with anti-TIGIT, HIV-1 Gag stimulation with anti-PD-L1, HIV-1 Gag stimulation with dual blockade (anti-TIGIT + anti-PD-L1) and a positive control (anti-CD3 + anti-CD28 Dynabeads). Graphs show compiled data showing variation in the frequency (%) of (B) CD8^+^ or (D) CD4^+^ T cell IL-2 in responses to HIV-1 Gag peptide pool with isotype control or mAb blockade; TIGIT blockade (left panel), PD-L1 blockade (middle panel), and dual blockade (right panel) (*n* = 16).
(TIF)

S6 Fig rhTIGIT amino acid sequence alignment, surface expression, γ-chain cytokine regulation and SIV-specific CD8^+^ T cell expression. (A) Alignment shows amino acid sequences of human TIGIT (Hu TIGIT) and Rhesus TIGIT (Rh TIGIT). Highlighted sequences indicate homology between human and rhesus TIGIT. Dashes indicate gaps in alignment. (B) Representative flow cytometry plots depict rhTIGIT expression frequency (%) on CD8^+^ T cells from PBMCs, LNs and spleen in representative non-infected and SIV-infected animals. (C) Representative flow cytometry plots depict rhTIGIT expression frequency (%) on naïve (N) (CD28^+^CD95^-^), effector memory (EM) (CD28^-^CD95^+^), and central memory (CM) (CD28^+^CD95^+^) cells from PBMCs, LNs and spleens in representative SIV-infected animals. Graphs show frequency (%) of rhTIGIT^+^ N, EM and CM CD8^+^ cells from SIV-infected PBMCs (open circle; *n* = 16), LNs (open square; *n* = 19), and spleens (open triangle; *n* = 10). P values were calculated with repeated-measures one-way ANOVA, followed by Tukey’s multiple comparisons test. (D) Graph shows compiled frequency (%) of rhTIGIT^+^ CD8^+^ T cells after stimulation with IL-2, IL-12 or IL-15 for six days. NS, no stimulation. P values were calculated with repeated-measures one-way ANOVA, followed by Tukey’s multiple comparisons test. (E) Representative flow cytometry plot showing secondary antibody only against CM9 tetramer staining to facilitate rhTIGIT gating. (F) Representative flow cytometry plot showing PD-1 FMO and secondary antibody only to facilitate rhTIGIT and PD-1 gating. (G) Representative flow cytometry plots of rhTIGIT and PD-1 expression on Mamu-A*01 SIV-Gag CM9 tetramer specific CD8^+^ T cells. Graphs show compiled data of rhTIGIT and PD-1 expression frequency (%) on Mamu-A*01 SIV-Gag CM9 tetramer specific CD8^+^ T cells (*n* = 4) from PBMC (open circle), LNs (open square), and spleen (open triangle). (H) Representative flow cytometry plots of rhTIGIT and PD-1 expression on Mamu-A*01 SIV-Tat SL8 tetramer specific CD8^+^ T cells. Graphs show rhTIGIT and PD-1 expression frequency (%) on Mamu-A*01 SIV-Tat SL8 tetramer specific CD8^+^ T cells (*n* = 4) from PBMC (open circle), LNs (open square), and spleen (open triangle).
(TIF)

S7 Fig Proliferative status of rhTIGIT expressing CD8^+^ T cells in SIV infection. (A) Representative flow cytometry plots depict Ki-67 and rhTIGIT expression in PBMCs and LNs from SIV-infected animals. (B) Graphs show frequency (%) of rhTIGIT^+^ Ki-67^+^ and rhTIGIT^-^ Ki-67^+^ CD8^+^ T cells in PBMCs (open circle; *n* = 11) and LNs (open square; *n* = 14) from SIV-infected animals. Wilcoxon matched-pairs signed- rank test was performed for statistical analysis (C) Representative flow cytometry plots depict Ki-67 and PD-1 expression in PBMCs and LNs from SIV-infected animals. (D) Graphs show frequency (%) of PD-1^+^ Ki-67^+^ and PD-1^-^ Ki-67^+^ CD8^+^ T cells in PBMCs (open circle; *n* = 6) and LNs (open square; *n* = 6) from SIV-infected animals. Wilcoxon matched-pairs signed- rank test was performed for statistical analysis.
(TIF)
