# Supplementary material for: TIGIT Marks Exhausted T Cells, Correlates with Disease Progression, and Serves as a Target for Immune Restoration in HIV and SIV Infection
Source: PLoS Pathog. 2016 Jan 7;12(1):e1005349. doi: 10.1371/journal.ppat.1005349 (PMC4704737; doi:10.1371/journal.ppat.1005349)
Supplement: S1 Text — (DOCX) [file ppat.1005349.s001.docx]

**Supplemental methods (NHP):**

**Animals**

Indian rhesus macaques (Macaca mulatta), housed at the Oregon National Primate Research Center and used in this study, were cared for according to the laws, regulations, and guidelines set forth by the U.S. Department of Agriculture (e.g., the Animal Welfare Act and its regulations, and the Animal Care Policy Manual), Institute for Laboratory Animal Research (e.g., Guide for the Care and Use of Laboratory Animals, 8th edition), Public Health Service, National Research Council, Centers for Disease Control and Infection, and the Association for Assessment and Accreditation of Laboratory Animal Care International. The Oregon Health and Science University Institutional Animal Care and Use Committee approved the research involving animals reported in this study. Animals were infected with SIVsmE660, SIVmac239, or SIVmac251 for other, unrelated projects.

**Antibodies and flow cytometric analysis**

The following directly conjugated Abs were obtained from BD Biosciences: Alexa Fluor 700–conjugated anti-CD3 (SP34-2), PE-CF594–conjugated anti-CD4 (L200), allophycocyanin- or allophycocyanin-H7–conjugated anti-CD8 (SK1), PE-Cy5–conjugated anti-CD95 (DX2), allophycocyanin-conjugated anti–IFN-γ (B27), and PE-conjugated anti–Ki-67 (B56). PE-conjugated anti–PD-1 (EH12.2H7), and PE-Cy7–conjugated CD28 (CD28.2) were obtained from Biolegend. PE-conjugated anit-TIGIT (MBSA43) was obtained from eBioscience. Allophycocyanin-conjugated Mamu-A*01 SIV Gag_181–189_ CM9 (CTPYDINQM) tetramer and allophycocyanin-conjugated Mamu-A*01 SIV Tat_28–35_ SL8 (STPESANL) tetramer were produced as described previously [[64](#_ENREF_64)]. An aqua amine reactive dye (Invitrogen) was used to exclude dead cells. In some experiments, cells were fixed in 2% paraformaldehyde (PFA), permeabilized with BD FACS permeabilizing solution 2 (BD Biosciences), and stained for Ki-67 and IFN-γ (BD Biosciences).

**Cloning and sequencing of rhesus macaque TIGIT**

Total RNA was purified from fresh rhesus PBMC using the AllPrep DNA/RNA kit (QIAGEN, Venlo, Limburg, The Netherlands). TIGIT was reverse transcribed with Superscript III One-Step RT-PCR System with Platinum Taq High Fidelity (Invitrogen) using primers 5’-ATGCGGTGGTGTCTCTTCC-3’ and 5’-CTACCCAGTCTCTGTGAAGAAGC-3’. The amplicon was purified from a 1% agarose gel and sequenced using the same primers, in addition to internal sequencing primers, 5’-ACTCAGCATTACGAATGGCCAG-3’ and 5’-ACTGGACAGGAAGAACAGATTCC-3’ to cover the 5’ and 3’ ends, respectively. CodonCode Aligner (CodonCode Corporation, Centerville, Massachusetts) was used to translate the DNA sequences, which were deposited into GenBank (KR534505).

**T cell stimulation and intracellular cytokine staining**

T cell stimulation and intracellular cytokine staining were performed similarly to a previous detailed description [[64](#_ENREF_64),[65](#_ENREF_65)]. Briefly, 5 × 10^5^ cryopreserved PBMCs or LN cells were incubated for one hour at 37 °C in 200 μl RPMI 1640 containing 10% bovine growth serum and antibiotics with anti-CD28, anti-CD49d, and 10 μM the synthetic peptide SIV Gag_181–189_CM9 (CTPYDINQM). In additional experiments, stimulated with 50 ng/mL of PMA and 1 μg/ml of ionomycin (Life Technologies). Then, 10 μg/ml brefeldin A (Sigma-Aldrich) was added, and the cells were incubated for an additional 6-8 hours at 37 °C. Cells were washed in buffer (PBS with 10% serum), stained for surface expression of CD3, CD4, and CD8 markers, and fixed in 2% PFA (Electron Microscopy Sciences) at 4 °C. Cells were then permeabilized in wash buffer containing 1% saponin and stained for the expression of cytokines IFN-γ (BD Biosciences). Stained cells were acquired on a custom four-laser BD Fortessa flow cytometer (BD Biosciences) with FACSDiva software and analyzed with FlowJo software (TreeStar).

References:

64. Mothe BR, Horton H, Carter DK, Allen TM, Liebl ME, et al. (2002) Dominance of CD8 responses specific for epitopes bound by a single major histocompatibility complex class I molecule during the acute phase of viral infection. J Virol 76: 875-884.

65. Vogel TU, Friedrich TC, O'Connor DH, Rehrauer W, Dodds EJ, et al. (2002) Escape in one of two cytotoxic T-lymphocyte epitopes bound by a high-frequency major histocompatibility complex class I molecule, Mamu-A*02: a paradigm for virus evolution and persistence? J Virol 76: 11623-11636.
