# Supplementary figures and images for: TIGIT Marks Exhausted T Cells, Correlates with Disease Progression, and Serves as a Target for Immune Restoration in HIV and SIV Infection
Source: PLoS Pathog. 2016 Jan 7;12(1):e1005349. doi: 10.1371/journal.ppat.1005349 (PMC4704737; doi:10.1371/journal.ppat.1005349)

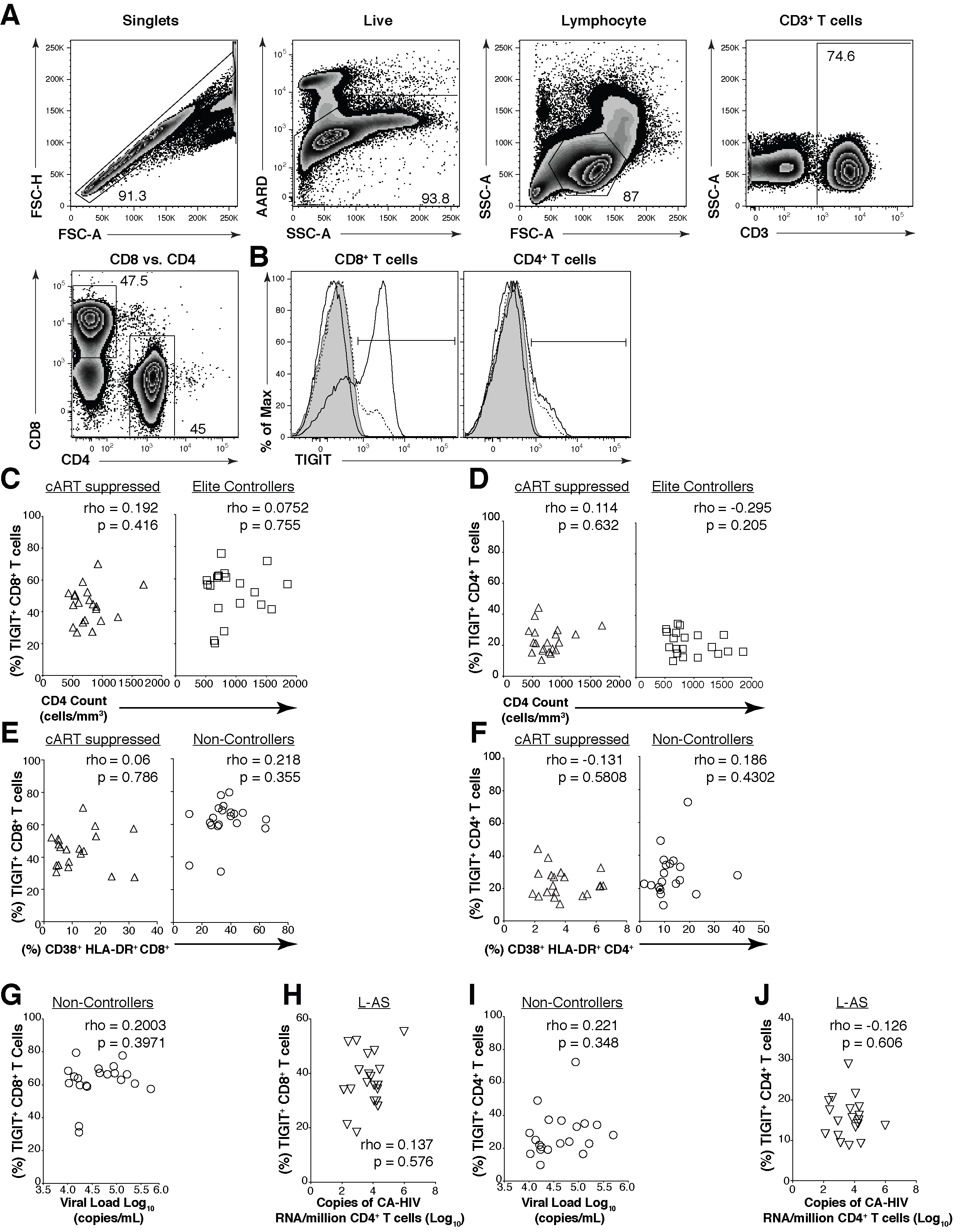

Supplement: S1 Fig — (A) Representative flow cytometry plots showing gating scheme to isolate CD8+ and CD4+ T cells. Gated on singlets, excluded dead cells, gated on lymphocytes, gated on CD3+ T cells, and gated on expression of CD8 or CD4. (B) Representative histograms of TIGIT Isotype, TIGIT FMO and TIGIT expression on CD8+ or CD4+ T cells (HIV-Infected thin solid line, HIV-Uninfected dashed line, TIGIT isotype control shaded, and TIGIT FMO thick solid line). Graphs show the association of the frequency (%) of (C) TIGIT+ CD8+ or (D) TIGIT+ CD4+ T cells against clinical CD4 count for cART suppressed (left panel, open triangle, n = 20) and elite controllers (right panel, open squares, n = 20). Graphs show the association of the frequency (%) of (E) TIGIT+ CD8+ or (F) TIGIT+ CD4+ T cells against T cell activation (% CD38+HLA-DR+) for cART suppressed (left panel, open triangle, n = 20) and non-controllers (right panel, open circles, n = 20). Graphs show the association of the frequency (%) of (G) TIGIT+ CD8+ or (I) TIGIT+ CD4+ T cells against viral load log10 (copies/ml) for non-controllers (open circles, n = 20). Graphs show the association of the frequency (%) of (H) TIGIT+ CD8+ or (J) TIGIT+ CD4+ T cells against copies of cell associated HIV RNA per million CD4+ T cells for L-AS (inverted open triangles, n = 19). Spearman’s rho tests were performed for correlations. (TIF) [file ppat.1005349.s002.tif]

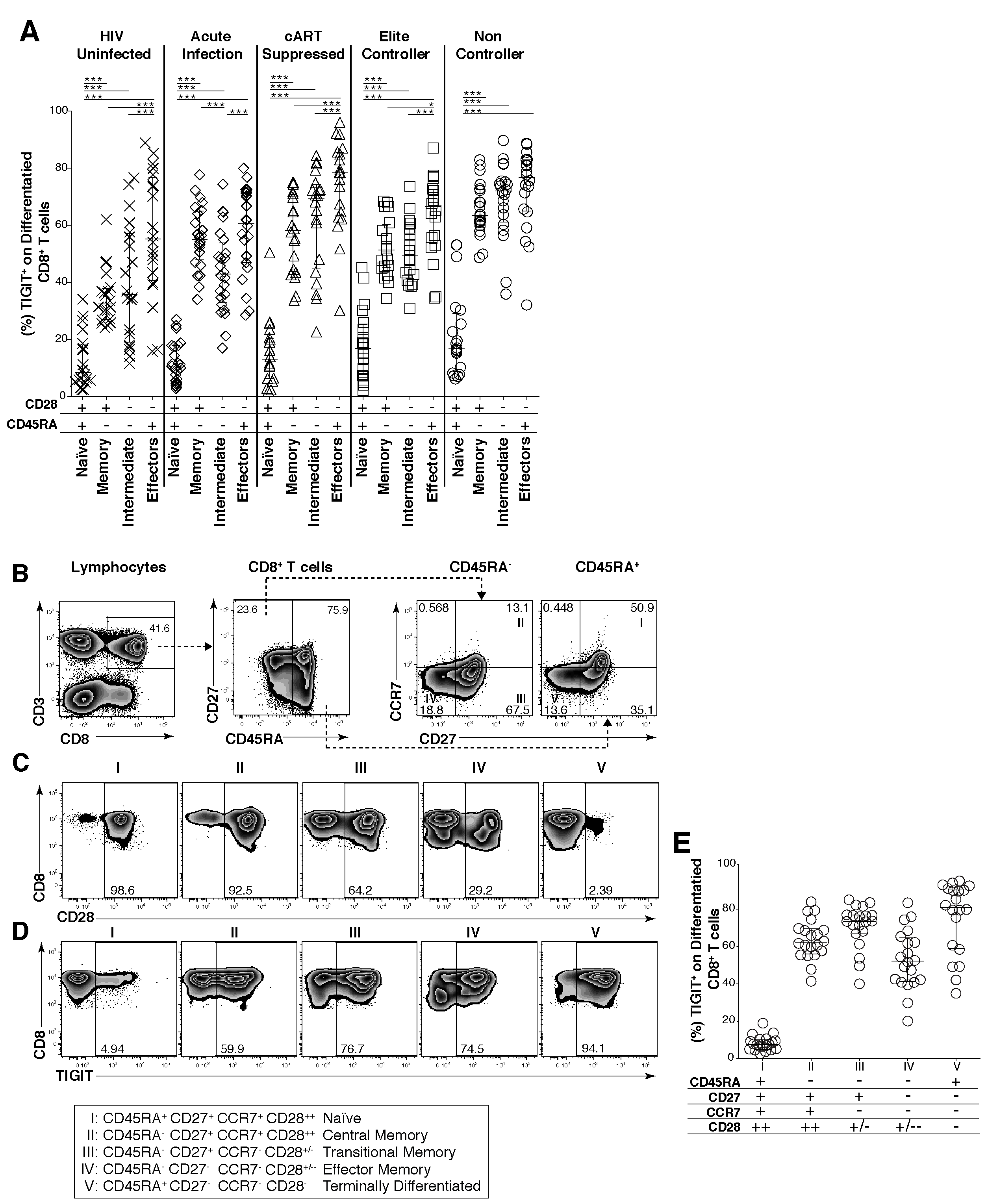

Supplement: S2 Fig — (A) Graph shows compiled frequency (%) of TIGIT expression on CD8+ T cells subsets grouped by disease category. HIV-Uninfected (X; n = 20), acute infected (AI; open diamond; n = 24), cART suppressed (AS; open triangle; n = 20), elite controller (EC; open square; n = 20), and non-controllers (NC; open circle; n = 20). Repeated-measures one-way ANOVA, followed by Tukey’s multiple comparisons test were used for comparison (*p < 0.05; **p < 0.01; ***p < 0.001). Cryopreserved PBMCs from chronically HIV-infected individuals were phenotyped for TIGIT expression on CD8+ T cell subsets. (B) Representative flow cytometry plots showing gating scheme to isolate CD8+ T cell subsets. Live lymphocytes gated for CD8+ T cells, subset into CD45RA+ and CD45RA-, further stratified by expression of CCR7 and CD27. (C) Representative flow cytometry plots showing CD28 expression on CD8+ T cell subsets. (D) Representative flow cytometry plots showing TIGIT expression on CD8+ T cell subsets. (E) Graph shows compiled frequency (%) of TIGIT expression on CD8+ T cell subsets (n = 20). (TIF) [file ppat.1005349.s003.tif]

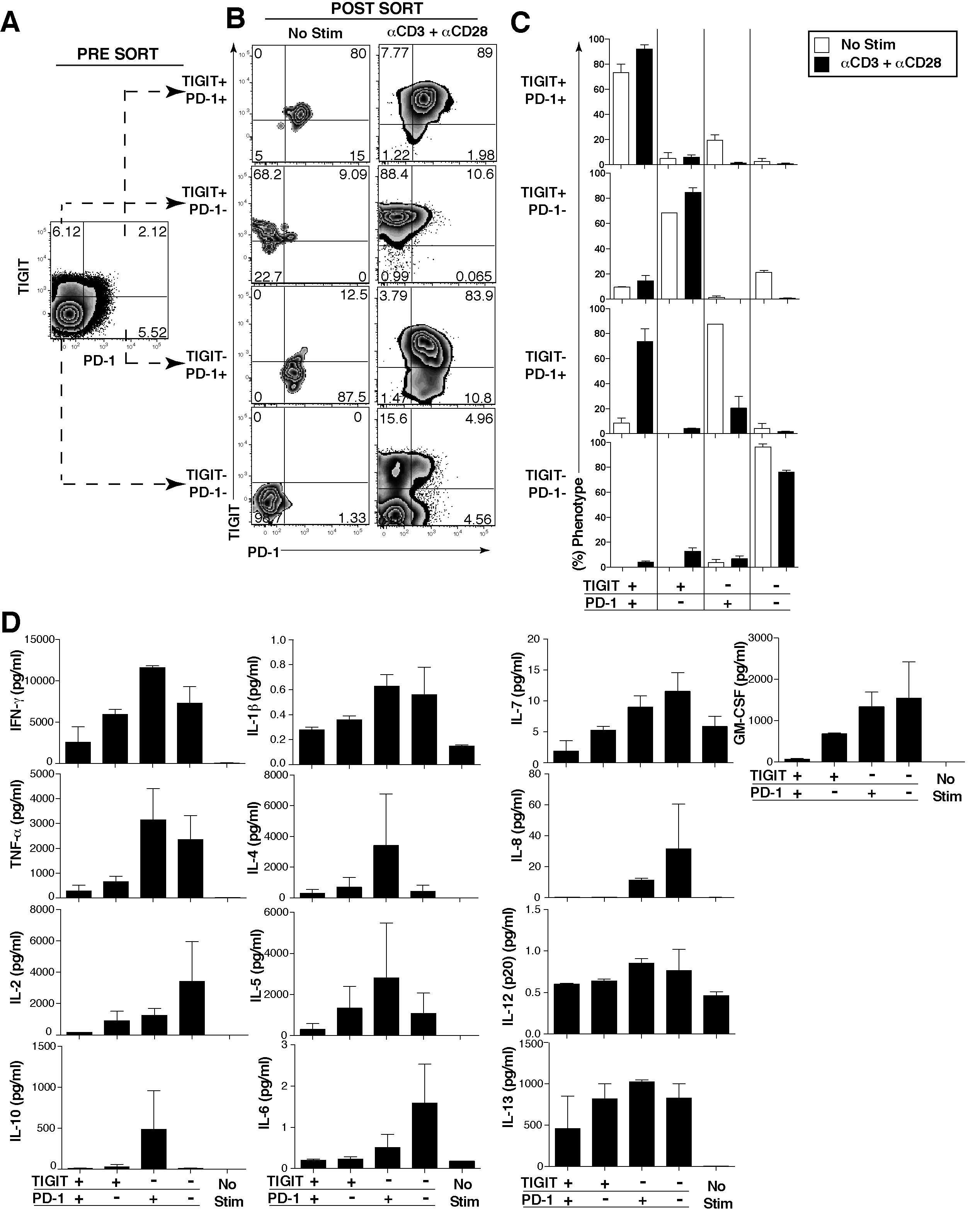

Supplement: S3 Fig — Ex vivo CD8+ T cells from chronically HIV-infected individuals were FACS sorted into populations according to their expression of TIGIT and PD-1. (A) Representative flow cytometry plot of TIGIT and PD-1 expression PRE-SORT. Gating was facilitated by isotype controls for TIGIT and PD-1. (B) Representative flow cytometry plots of CD8+ T cells sorted into TIGIT+PD-1+, TIGIT+PD-1-, TIGIT-PD-1+, and TIGIT-PD-1-. No stimulation (left panel) and stimulated with anti-CD3 + anti-CD28 Dyanbeads for 48 hours (right panel). (C) Graphs show compiled data of phenotypes of sorted populations with no stimulation (open box) and anti-CD3 + anti-CD28 Dyanbeads (filled box) (n = 2). Supernatants were harvested and cytokine production was assessed 48 hours post anti-CD3 + anti-CD28 stimulation by high sensitivity multiplex bead array. (D) Graphs show concentrations of cytokines produced from sorted populations. (TIF) [file ppat.1005349.s004.tif]

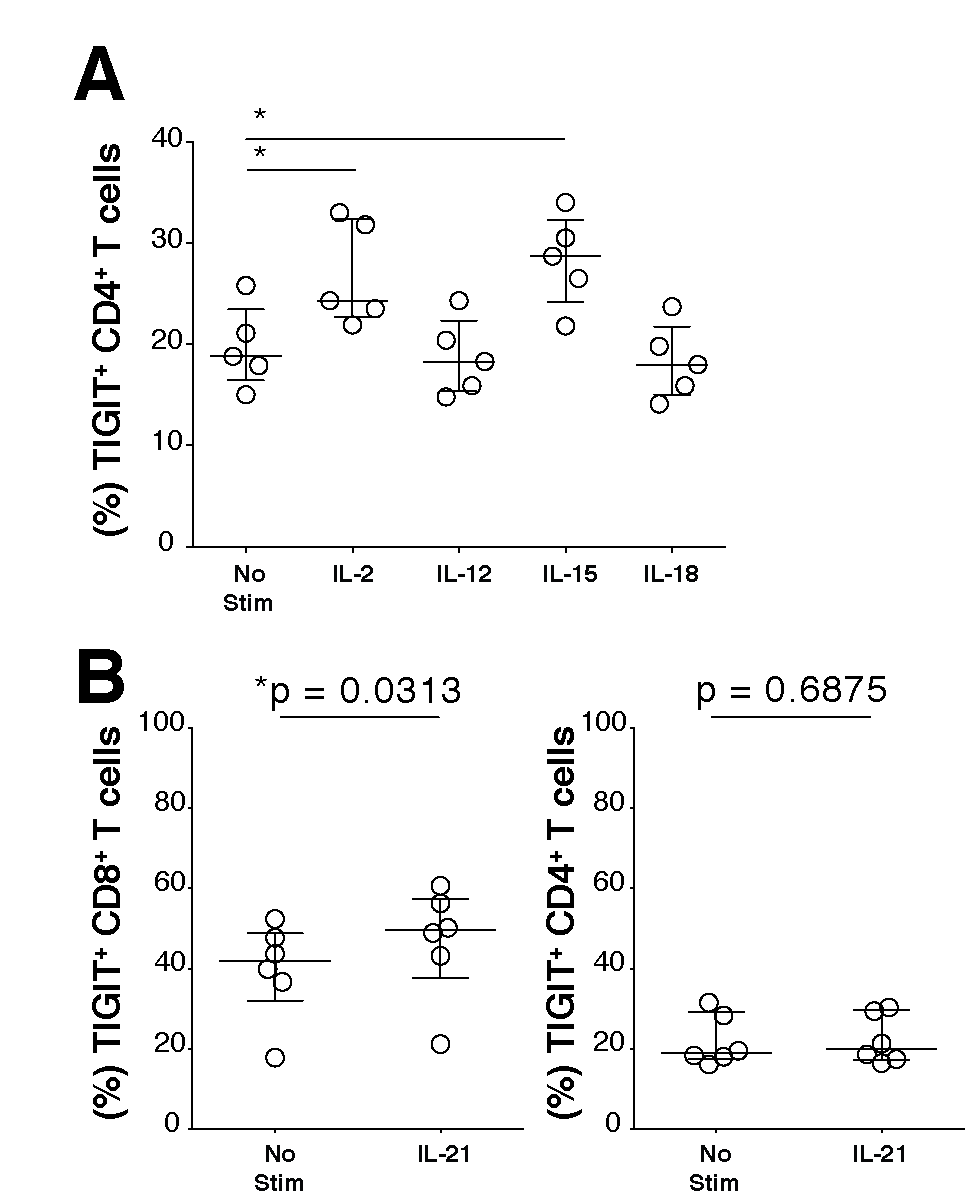

Supplement: S4 Fig — (A) Compiled data of HIV-Infected individuals (open circle; n = 8) TIGIT expression frequency (%) on CD4+ T cells with or without cytokine stimulation for six days. P values were calculated with repeated-measures one-way ANOVA, followed by Tukey’s multiple comparisons test (*p < 0.05). (B) Compiled data of HIV-Infected individuals (open circle; n = 6) TIGIT expression frequency (%) on CD8+ T cells (right panel) and CD4+ T cells (left panel) after six days of IL-21 stimulation (n = 6). P values were calculated by Wilcoxon matched-pairs signed ranked test. (TIF) [file ppat.1005349.s005.tif]

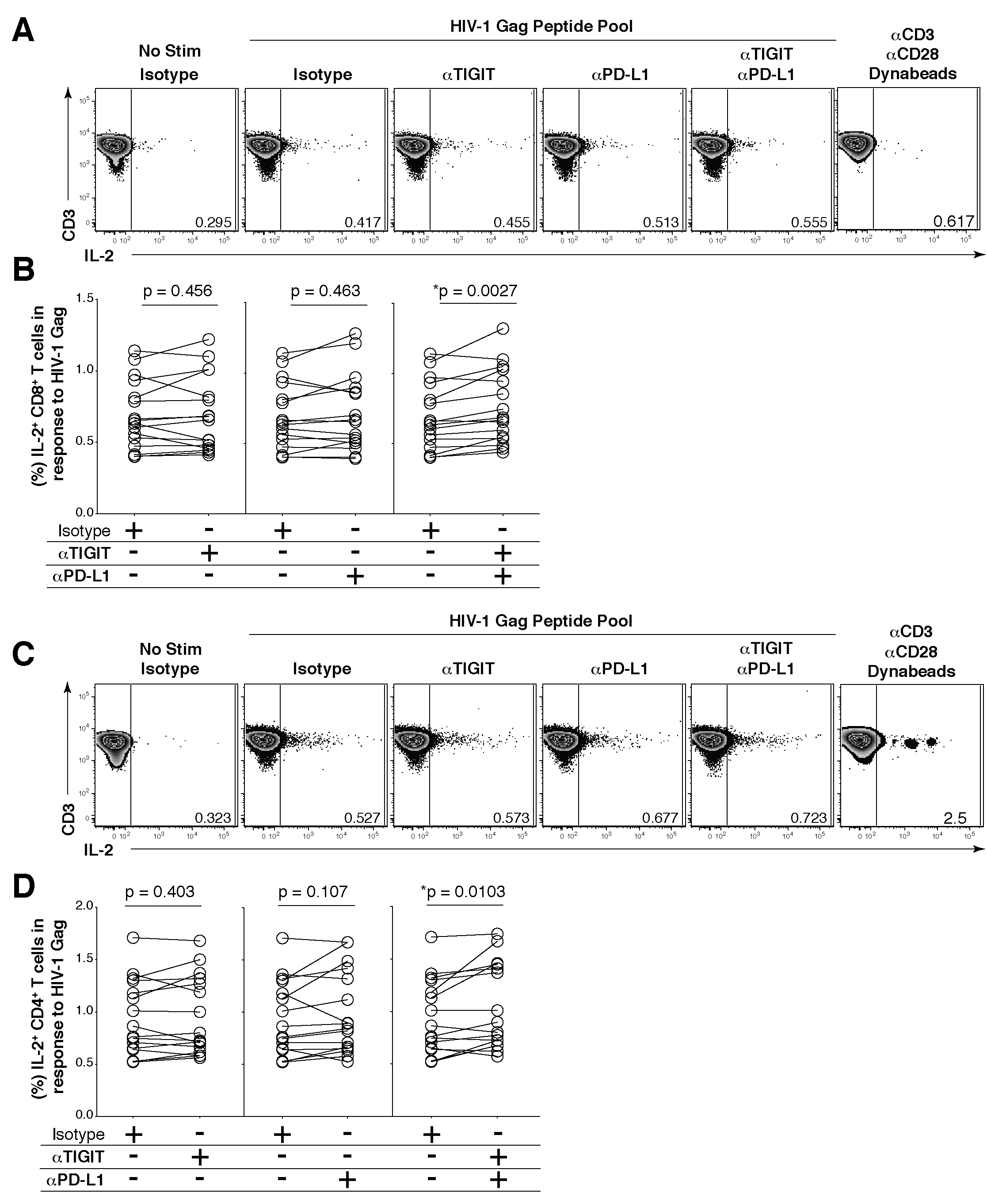

Supplement: S5 Fig — Ex vivo PBMCs from chronically HIV-infected individuals were stimulated with HIV Gag peptide pool in the presence of mAb blocking antibodies. Representative flow cytometry plots gated on (A) CD8+ or (C) CD4+ T cells, showing IL-2 responses from an HIV-infected individual. No HIV-1 Gag stimulation with an isotype control, HIV-1 Gag stimulation with an isotype control, HIV-1 Gag stimulation with anti-TIGIT, HIV-1 Gag stimulation with anti-PD-L1, HIV-1 Gag stimulation with dual blockade (anti-TIGIT + anti-PD-L1) and a positive control (anti-CD3 + anti-CD28 Dynabeads). Graphs show compiled data showing variation in the frequency (%) of (B) CD8+ or (D) CD4+ T cell IL-2 in responses to HIV-1 Gag peptide pool with isotype control or mAb blockade; TIGIT blockade (left panel), PD-L1 blockade (middle panel), and dual blockade (right panel) (n = 16). (TIF) [file ppat.1005349.s006.tif]

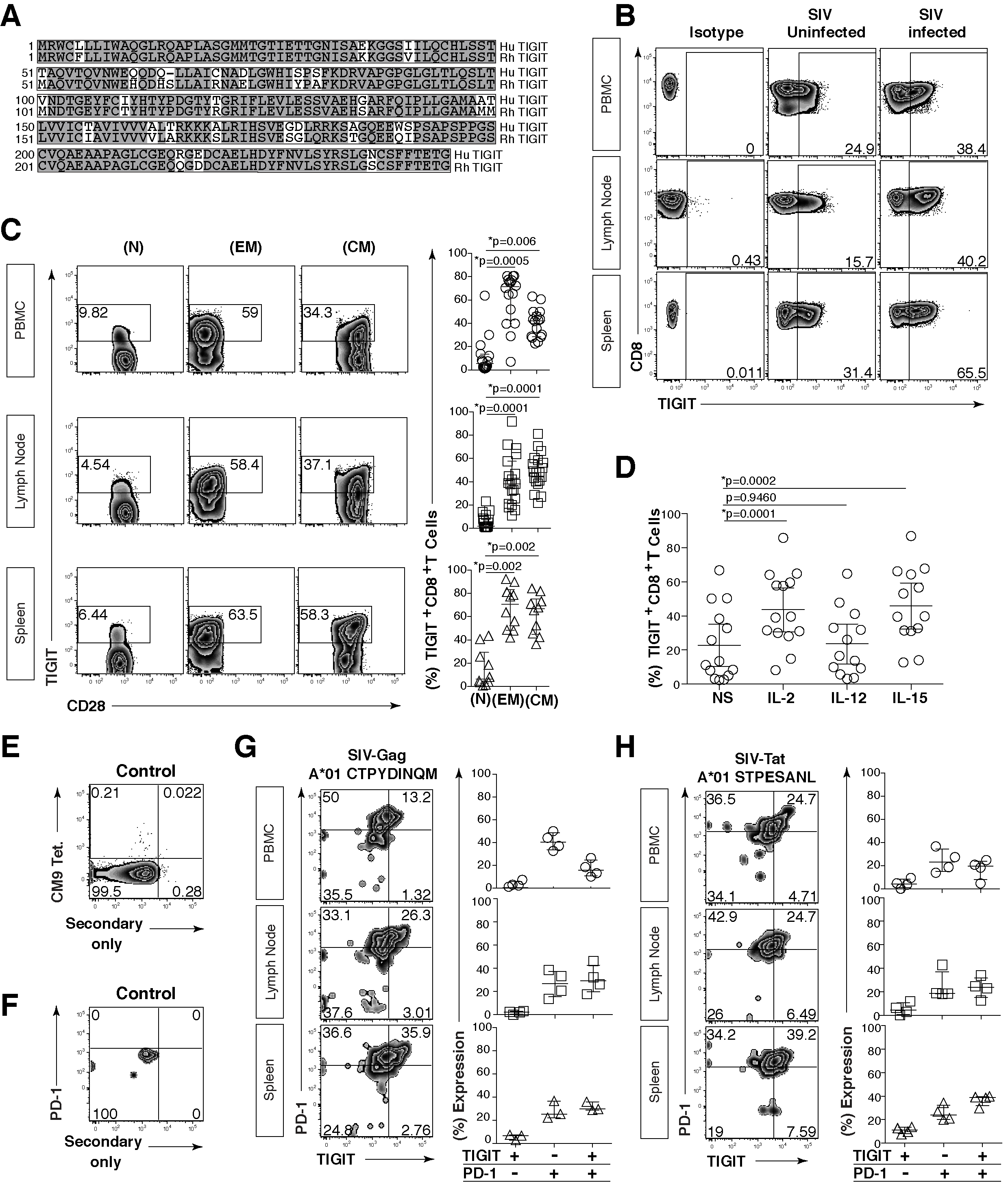

Supplement: S6 Fig — (A) Alignment shows amino acid sequences of human TIGIT (Hu TIGIT) and Rhesus TIGIT (Rh TIGIT). Highlighted sequences indicate homology between human and rhesus TIGIT. Dashes indicate gaps in alignment. (B) Representative flow cytometry plots depict rhTIGIT expression frequency (%) on CD8+ T cells from PBMCs, LNs and spleen in representative non-infected and SIV-infected animals. (C) Representative flow cytometry plots depict rhTIGIT expression frequency (%) on naïve (N) (CD28+CD95-), effector memory (EM) (CD28-CD95+), and central memory (CM) (CD28+CD95+) cells from PBMCs, LNs and spleens in representative SIV-infected animals. Graphs show frequency (%) of rhTIGIT+ N, EM and CM CD8+ cells from SIV-infected PBMCs (open circle; n = 16), LNs (open square; n = 19), and spleens (open triangle; n = 10). P values were calculated with repeated-measures one-way ANOVA, followed by Tukey’s multiple comparisons test. (D) Graph shows compiled frequency (%) of rhTIGIT+ CD8+ T cells after stimulation with IL-2, IL-12 or IL-15 for six days. NS, no stimulation. P values were calculated with repeated-measures one-way ANOVA, followed by Tukey’s multiple comparisons test. (E) Representative flow cytometry plot showing secondary antibody only against CM9 tetramer staining to facilitate rhTIGIT gating. (F) Representative flow cytometry plot showing PD-1 FMO and secondary antibody only to facilitate rhTIGIT and PD-1 gating. (G) Representative flow cytometry plots of rhTIGIT and PD-1 expression on Mamu-A*01 SIV-Gag CM9 tetramer specific CD8+ T cells. Graphs show compiled data of rhTIGIT and PD-1 expression frequency (%) on Mamu-A*01 SIV-Gag CM9 tetramer specific CD8+ T cells (n = 4) from PBMC (open circle), LNs (open square), and spleen (open triangle). (H) Representative flow cytometry plots of rhTIGIT and PD-1 expression on Mamu-A*01 SIV-Tat SL8 tetramer specific CD8+ T cells. Graphs show rhTIGIT and PD-1 expression frequency (%) on Mamu-A*01 SIV-Tat SL8 tetramer specific CD8+ T cells (n [file ppat.1005349.s007.tif]

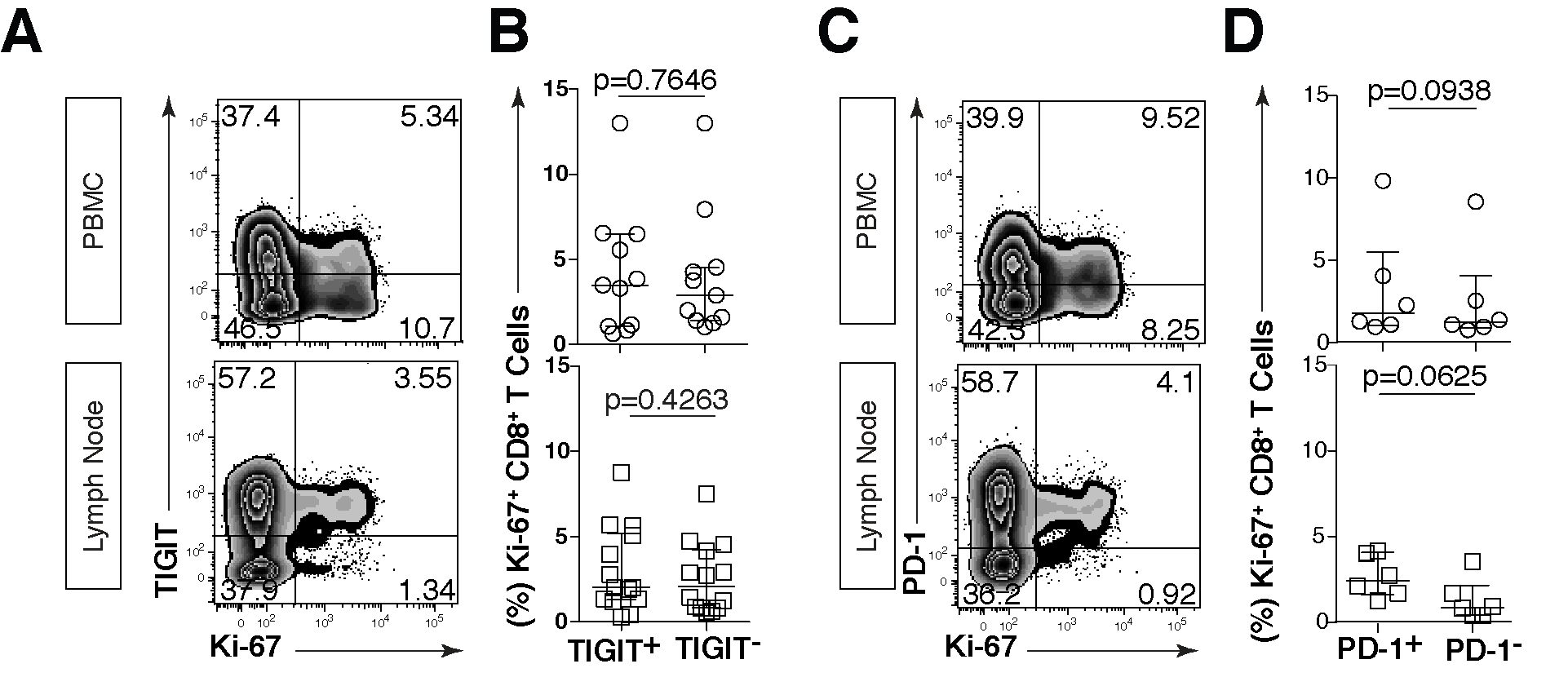

Supplement: S7 Fig — (A) Representative flow cytometry plots depict Ki-67 and rhTIGIT expression in PBMCs and LNs from SIV-infected animals. (B) Graphs show frequency (%) of rhTIGIT+ Ki-67+ and rhTIGIT- Ki-67+ CD8+ T cells in PBMCs (open circle; n = 11) and LNs (open square; n = 14) from SIV-infected animals. Wilcoxon matched-pairs signed- rank test was performed for statistical analysis (C) Representative flow cytometry plots depict Ki-67 and PD-1 expression in PBMCs and LNs from SIV-infected animals. (D) Graphs show frequency (%) of PD-1+ Ki-67+ and PD-1- Ki-67+ CD8+ T cells in PBMCs (open circle; n = 6) and LNs (open square; n = 6) from SIV-infected animals. Wilcoxon matched-pairs signed- rank test was performed for statistical analysis. (TIF) [file ppat.1005349.s008.tif]
